# Recognition of extracellular DNA by type IV pili promotes biofilm formation by *Clostridioides difficile*

**DOI:** 10.1016/j.jbc.2022.102449

**Published:** 2022-09-03

**Authors:** Leslie A. Ronish, Ben Sidner, Yafan Yu, Kurt H. Piepenbrink

**Affiliations:** 1 Department of Biochemistry, University of Nebraska-Lincoln, Lincoln, Nebraska, USA; 2 Department of Food Science and Technology, University of Nebraska-Lincoln, Lincoln, Nebraska, USA; 3 Department of Chemistry, University of Nebraska-Lincoln, Lincoln, Nebraska, USA; 4 Nebraska Food for Health Center, University of Nebraska-Lincoln, Lincoln, Nebraska, USA; 5 Center for Integrated Biomolecular Communication, University of Nebraska-Lincoln, Lincoln, Nebraska, USA

**Keywords:** bacterial biofilm, type IV pili, C. difficile, protein DNA interactions, extracellular matrix, structural biology, X-ray crystallography, adhesion, surface motility, CLSM, confocal laser scanning microscopy, eDNA, extracellular DNA, EtBr, ethidium bromide, MBP, maltose-binding protein, T4F, type IV filaments, T4P, type IV pilus, TCP, toxin-coregulated pilus

## Abstract

*Clostridioides difficile* is a Gram-positive *bacillus*, which is a frequent cause of gastrointestinal infections triggered by the depletion of the gut microbiome. Because of the frequent recurrence of these infections after antibiotic treatment, mechanisms of *C. difficile* persistence and recurrence, including biofilm formation, are of increasing interest. Previously, our group and others found that type IV pili, filamentous helical appendages polymerized from protein subunits, promoted microcolony and biofilm formation in *C. difficile*. In Gram-negative bacteria, the ability of type IV pili to mediate bacterial self-association has been explained through interactions between the pili of adjacent cells, but type IV pili from several Gram-negative species are also required for natural competence through DNA uptake. Here, we report the ability of two *C. difficile* pilin subunits, PilJ and PilW, to bind to DNA *in vitro*, as well as the defects in biofilm formation in the *pilJ* and *pilW* gene-interruption mutants. Additionally, we have resolved the X-ray crystal structure of PilW, which we use to model possible structural mechanisms for the formation of *C. difficile* biofilm through interactions between type IV pili and the DNA of the extracellular matrix. Taken together, our results provide further insight into the relationship between type IV pilus function and biofilm formation in *C. difficile* and, more broadly, suggest that DNA recognition by type IV pili and related structures may have functional importance beyond DNA uptake for natural competence.

*Clostridioides difficile* is a Gram-positive, spore-forming anaerobic *bacillus*, which is a common cause of gastrointestinal infections, particularly after the use of oral antibiotics or other treatments which reduce the diversity of the gut microbiome (1). These infections are treatable with one of several oral antibiotic treatments, but in 20 to 30% of cases, the infection reappears after the cessation of antibiotic treatment (2, 3). Several mechanisms have been proposed for this recurrence, including the possibility of a reservoir of *C. difficile* biofilm which persists through the course of antibiotics (4, 5). Notably, this same pattern of recurrence has been observed for other bacterial infections in which the pathogen is known to form biofilm, including *Pseudomonas aeruginosa* infections which form biofilms in the lung (6).

The ability of *C. difficile* to form biofilms *in vitro* is well established from work by our group and others (7, 8, 9, 10, 11). These bacterial communities contain populations resistant to antimicrobials (12) and may contribute to the recurrence of *C. difficile* infections either through persistent populations of vegetative cells or through increased adherence of spores. Biofilm formation *in vivo* by *C. difficile* has been observed in animal models of infection (13). The formation of *C. difficile* biofilms *in vitro* is typically studied in monoculture, but *in vivo*, interactions with other bacterial species, either pathogens or commensal members of the gut microbiome, may play an important role (14, 15), either cooperatively or competitively.

Bacterial biofilms are composed not only of bacterial cells but an extensive extracellular matrix composed of polysaccharides, extracellular DNA, and polypeptides (16, 17, 18, 19), either as monomers (20) or as surface assemblies (21, 22). Previously, our group and others have reported a defect in *in vitro* biofilm formation for mutations in the type IV pilus (T4P) system in *C. difficile* (7, 8, 9). T4P are helical fibers extended from the cell surface through the noncovalent polymerization of thousands of protein subunits called pilins (23). All T4P fibers appear to be heterogeneous, incorporating multiple subunit types; one subunit, the major pilin, typically predominates, making up over 99% of the fiber, with other subunits incorporated either at the tip (24, 25) or sporadically throughout the fiber (26, 27). We originally hypothesized that *C. difficile* T4P promoted bacterial aggregation/assembly based on the structural similarity of the *C. difficile* T4P major pilin, PilA1, to TcpA, the major pilin subunit of the toxin-coregulated pilus (TCP) of *Vibrio cholerae* (26, 28).

T4P systems can be found in a wide variety of Gram-negative (29, 30) and Gram-positive bacteria (23, 31). These adhesive fibers can be extended through a cytoplasmic hexameric ATPase (PilB) and retracted quickly with considerable force (32) through a homologous ATPase (PilT); the combination of adhesion and retraction allows them to mediate several distinct physiological processes: host-cell adhesion (25, 33), surface (twitching) motility (34), horizontal gene transfer through DNA uptake (33, 35, 36), and the formation of microcolonies/biofilms through bacterial aggregation (37, 38). None of these four functions is found universally in T4P+ bacteria; although T4P systems in some organisms such as *Acinetobacter baumannii* (39, 40, 41, 42) and *P. aeruginosa* (43, 44, 45, 46) show all four. The functions of T4P systems in Gram-positive bacteria are less well understood, but considerable progress has been made recently in characterizing the T4P of *Clostridium perfringens* (47, 48, 49), *Streptococcus sanguinis* (50, 51, 52, 53, 54, 55, 56), and *C. difficile* (7, 8, 9, 26, 57). In *C. difficile*, T4P-deficient mutants show defects in surface motility (8) and biofilm formation (8, 9) but their role in adhesion to host is more complicated (57) and no experimental conditions have been reported in which *C. difficile* exhibits natural competence. *C. difficile* T4P genes are upregulated both in strains 630 and R20291 but the pattern differs between the two strains (8, 9, 58).

Previously, we reported that *C. difficile* T4P promote biofilm formation through an unknown mechanism (9). Here, we have tested the hypothesis that interactions between *C. difficile* T4P and extracellular DNA contribute to biofilm formation by measuring the ability of recombinantly expressed *C. difficile* T4P subunits to bind DNA. We have found that two pilin subunits, PilJ and PilW, bind DNA *in vitro*. Based on these results, we have measured the ability of gene-interruption mutants of *pilJ* and *pilW* to form bacterial biofilms and resolved the x-ray crystal structure of PilW to probe the mechanisms by which these subunits adhere to DNA and how these structurally distinct proteins are incorporated into the pilus fiber.

## Results

### pilJ and pilW gene-interruption mutants show defects in biofilm formation

**Figure 1:**
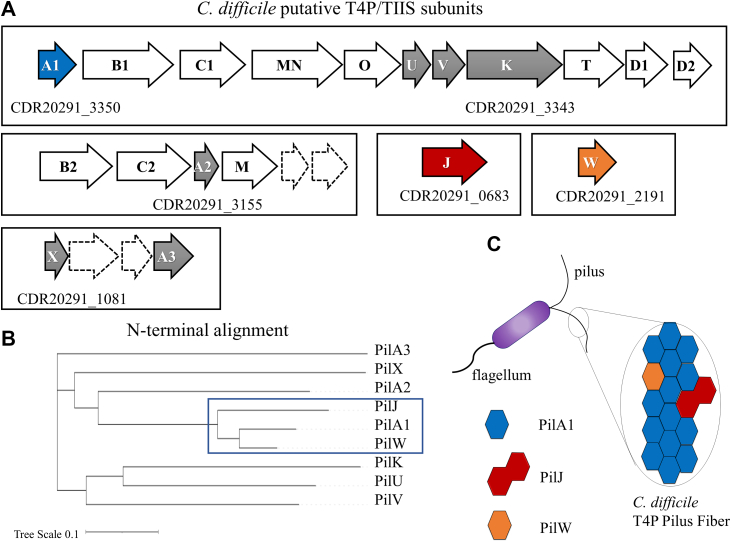
***Clostridioides difficile* type IV pilus subunits.***A*, putative subunits for type IV pilus and type II secretion systems in *C. difficile* R20291 (identified by their N-terminal sequences) and gene clusters containing putative pilins are shown. For at least one pilin gene in each cluster, the NCBI gene identifier is noted below. *B*, alignment of the nine subunits based on the N-terminal amino acid sequence (pre-pilin leader sequence and α1-N helix, ∼30 residues). *C*, proposed scheme for heterologous subunit incorporation.

Although T4P are primarily composed of single subunit, all known T4P systems encode genes for multiple protein subunits (30). While most of the research into the incorporation of minor T4P (and type II secretion) subunits has focused on initiator pilins or initiation complexes at tip (56, 59, 60, 61, 62), increasingly, evidence from our own group and others (27) indicates that some minor subunits are incorporated sporadically throughout the length of the T4P fiber, including *C. difficile* PilJ (26). In the UK epidemic strain R20291, the primary *C. difficile* T4P operon includes all genes, which have been identified as necessary for pilus polymerization, including an extension ATPase (*pilB1),* as well as four pilin genes, including *pilA1* (the major subunit). Two other pilin gene clusters contain a total of three pilin genes and a further two, *pilJ* and *pilW* can be identified outside of any such gene cluster (31, 63) (Fig. 1*A*). For the majority of these subunits, their role in the formation of pili or pseudopili remains unclear, but the presence of multiple pilB, pilC, pilM(N), and pilD genes suggest that multiple structures exist. While sequence similarity between these genes is largely restricted to the conserved N-terminal helix, that region is essential for interactions between subunits in T4P (64) and may be able to identify subunits capable of incorporation into a pilus fiber composed primarily of PilA1. Figure 1*B* shows a dendrogram created from the N-terminal sequences of the nine subunits, the N-terminal tag which is removed by the peptidase pilD and the α1-N transmembrane helix. Two clusters, (PilA1, PilJ, PilW) and (PilU, PilV, PilK), were identified, suggesting that PilW and PilJ could be incorporated sporadically along the pilus in the place of PilA1 subunits (Fig. 1*C*), while the other six genes may be incorporated into a tip complex (PilU, PilV, PilK) or a TIIS-analog (PilA2, PilX, PilA3) (31).

T4P mutants defective for T4P synthesis show a reduction in biofilm formation in multiple *in vitro* models. Previously, we found the greatest defect in a *pilA1* gene-interruption mutant at early time points (24 h), becoming less pronounced at longer time points (7 days) using a static growth model with biomass measured by Confocal Laser Scanning Microscopy (CLSM) (9). Similarly, Purcell *et al.* found that *pilA1* and *pilB1* mutants were defective in biofilm production in static assays over 24 to 48 h, quantified by crystal violet staining, as well as in surface motility (8). To probe the structural basis for the promotion of biofilm by *C. difficile* T4P, we measured the ability of *pilJ* and *pilW* mutants to form biofilm *in vitro* using models similar to those described above.

**Figure 2:**
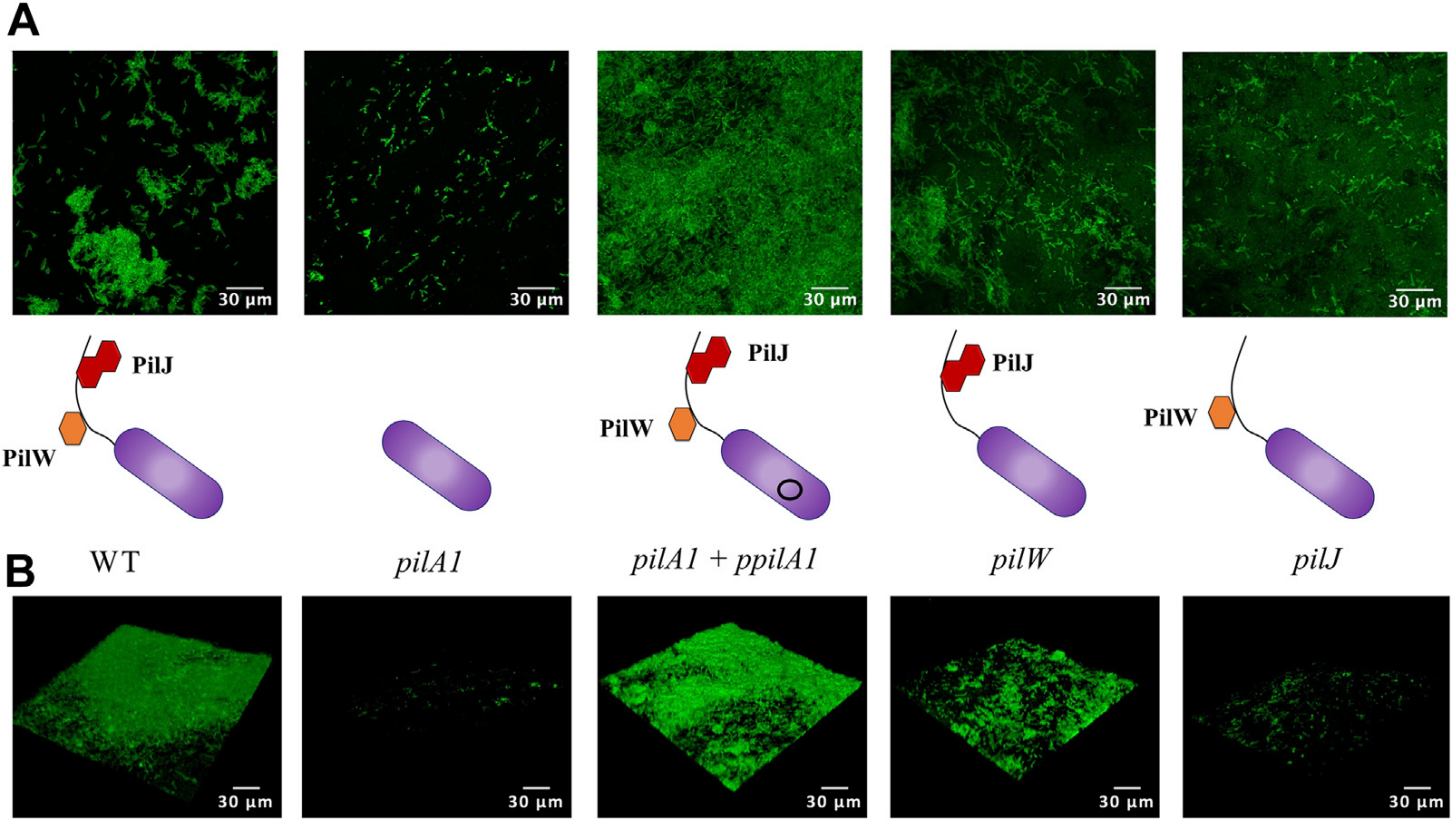
**Biofilm formation by *Clostridioides difficile* R20291 mutants.***A*, *top-down* CLSM images of *in vitro* biofilms of *C. difficile* R202091 WT and pilus mutants; *pilA1*, *pilJ,* and *pilW* are gene-interruption mutants of their respective genes, *pilA1* + ppilA1 is the *pilA1* mutant complemented with a plasmid containing the *pilA1* gene. *B*, 3D reconstructions of biofilms from z-stacks. CLSM, confocal laser scanning microscopy.

**Figure 3:**
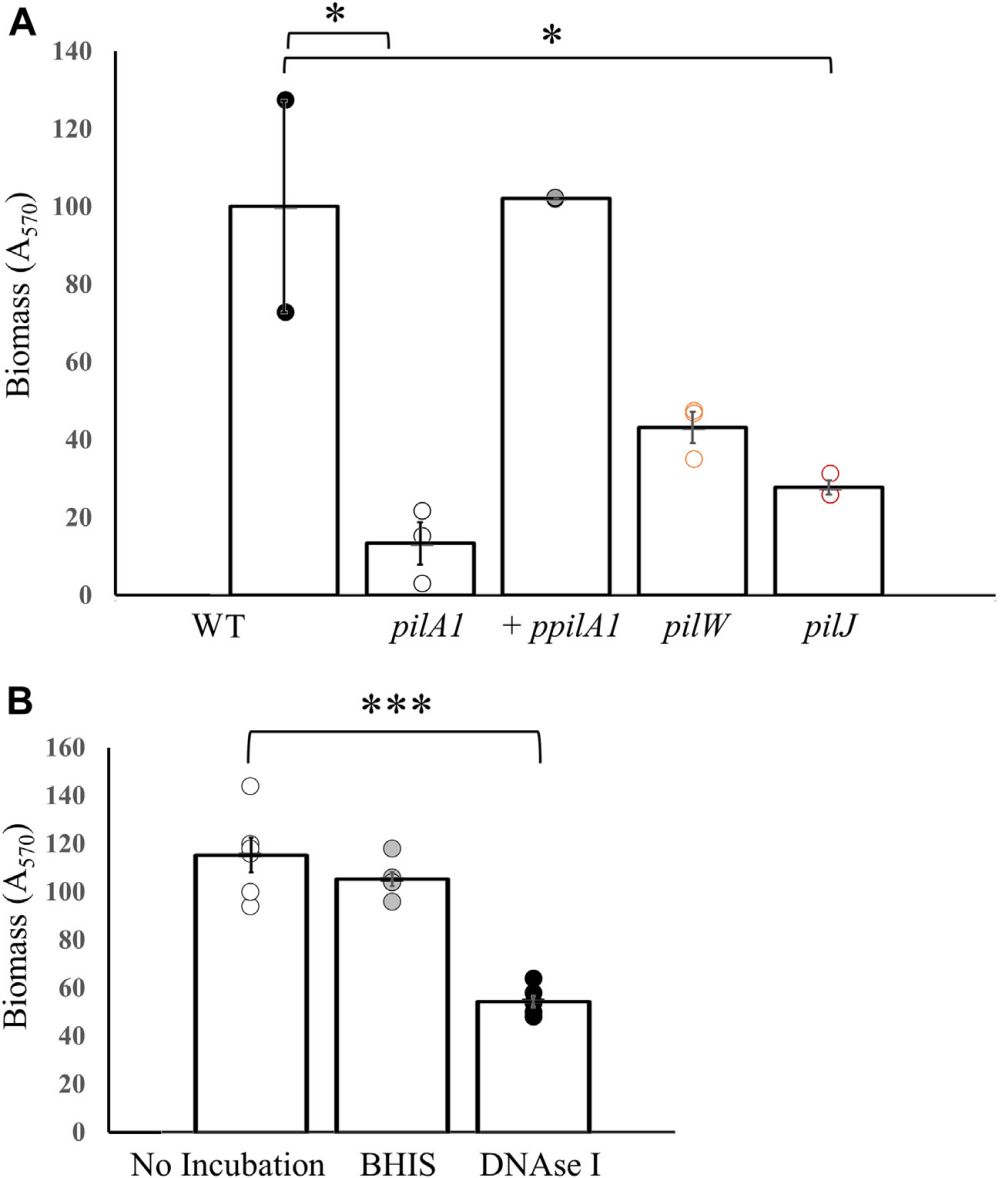
**Quantification of *Clostridioides difficile* biofilm formation.***A*, quantification of biomass by *crystal violet* staining. *B*, biomass recovered after treatment incubation with either BHIS media or BHIS with recombinant DNAse.

Figure 2 shows the results of the static biofilm formation assays in which the bacteria were grown in BHIS on metal coupons to facilitate attachment, with growth analyzed by CLSM. Data are shown for WT R20291, the *pilA1* gene-interruption mutant, and its complement (using the p84151 plasmid) as well as *pilJ* and *pilW* gene-interruption mutants. Figure 2*A* shows top-down images of the surface, while Figure 2*B* shows 3D reconstructions from z-stacks of confocal images. The *pilJ* and *pilW* mutants show phenotypes for biofilm formation intermediate between the WT and the *pilA1* mutant, suggesting that these strains produce T4P with a functional defect. These results fit a model in which PilJ and PilW are accessory subunits which functionalize T4P without being essential for their synthesis. In Figure 3*A*, we quantify this biofilm formation using crystal violet staining of horizontal biofilms, which agrees well with the CLSM data. In this assay, *pilJ* forms 70% less biofilm than WT R20291 and *pilW* 50% less; both more than the *pilA1* mutant, implying that some T4P function is retained in these mutants. We note that Tremblay *et al.* found *pilW* to be upregulated in biofilm but found no defect in biofilm formation for a *pilW* mutant of strain 630 (58); we attribute this difference to differential expression of *pilW* between R20291 and 630 (8). Measurements of twitching motility (a type of T4P-dependent surface motility) showed defects in the *pilA1*, *pilJ*, and *pilW* mutants with incomplete recovery for a *pilA1* complement (Fig. S1).

### Extracellular DNA is essential for the stability of *C. difficile* biofilm

To determine the degree to which extracellular DNA (eDNA) in the biofilm matrix stabilizes these *in vitro* biofilms, we incubated mature biofilms in fresh media (BHIS) with or without the addition of recombinant DNAse I. In Figure 3*B*, the results show that a 15 min DNAse treatment reduced the biomass by approximately 50% when compared with media alone (*p* = 0.00001123). These results are similar to those previously described for *in vitro* bacterial biofilms (65, 66), including Dawson *et al.* (67) which found that the addition of DNAse potentiated the activity of vancomycin against *C. difficile* biofilms.

### X-ray crystal structure of PilW

The incorporation of PilJ into *C. difficile* T4P has profound structural implications because PilJ has an unusual dual-pilin fold, in which the N-terminal domain is incorporated into the pilus in a manner similar to PilA1, but the C-terminal domain extends out from the pilus to provide a distinct interaction surface (26). However, sequence analysis of PilW led us to expect a single soluble domain more similar to PilA1. To understand how the incorporation of PilW would alter the T4P fiber, we resolved the X-ray crystal structure of PilW, as a C-terminal fusion to maltose-binding protein (MBP) at 2.5 Å resolution (Table 1). MBP crystallizes readily and can serve as a molecular chaperone to increase the propensity for crystallization (68), Fig. S2 shows the asymmetric unit of the MBP-PilW crystal, where contracts between MBP and PilW predominate. Fitting with the notion that MBP serves as a sort of molecular chaperone, the relative B-factors are lower for MBP than the majority of PilW, particularly the PilW surface loops (Fig. S3). However, the electron density for PilW is sufficient to build the entire structure through residue 152 in two of the four chains, leaving gaps in the αβ-loop in the other two as described below (Fig. S4).

**Table 1:** X-ray crystal structure of MBP-PilW^a^

Wavelength (Å)	1.033
Resolution range (Å)	38.33–2.487 (2.576–2.487)
Space group	P 1
Unit cell (Å)	65.3257 Å 81.7962 Å 102.964 Å, 92.441° 90.9457° 113.367°
Total reflections	150,043 (10,852)
Unique reflections	64,217 (6634)
Multiplicity	2.3 (2.4)
Completeness (%)	93.50 (96.17)
Mean I/sigma(I)	6.8 (2.5)
Wilson B-factor (Å^2^)	41.35
R-merge	0.088 (0.221)
R-meas	0.122 (0.301)
R-pim	0.060 (0.207)
CC1/2	0.993 (0.901)
CC^a^	0.999 (0.987)
Reflections used in refinement	64,180 (6634)
Reflections used for R-free	3188 (298)
R-work	0.1864 (0.2321)
R-free	0.2182 (0.2563)
Number of nonhydrogen atoms	15,965
macromolecules	15,236
ligands	104
solvent	625
Protein residues	1984
RMS(bonds) (Å)	0.004
RMS(angles) (˚)	0.78
Ramachandran favored (%)	95.09
Ramachandran allowed (%)	4.31
Ramachandran outliers (%)	0.61
Rotamer outliers (%)	1.73
Clash score	4.19
Average B-factor (Å^2^)	61.99
MBP (residues 0–370)	49.16
PilW (residues 1026–1152)	102.1
macromolecules	62.32
ligands	72.23
solvent	52.20
Number of TLS groups	32

a Values in parentheses are for the highest resolution shell.

**Figure 4:**
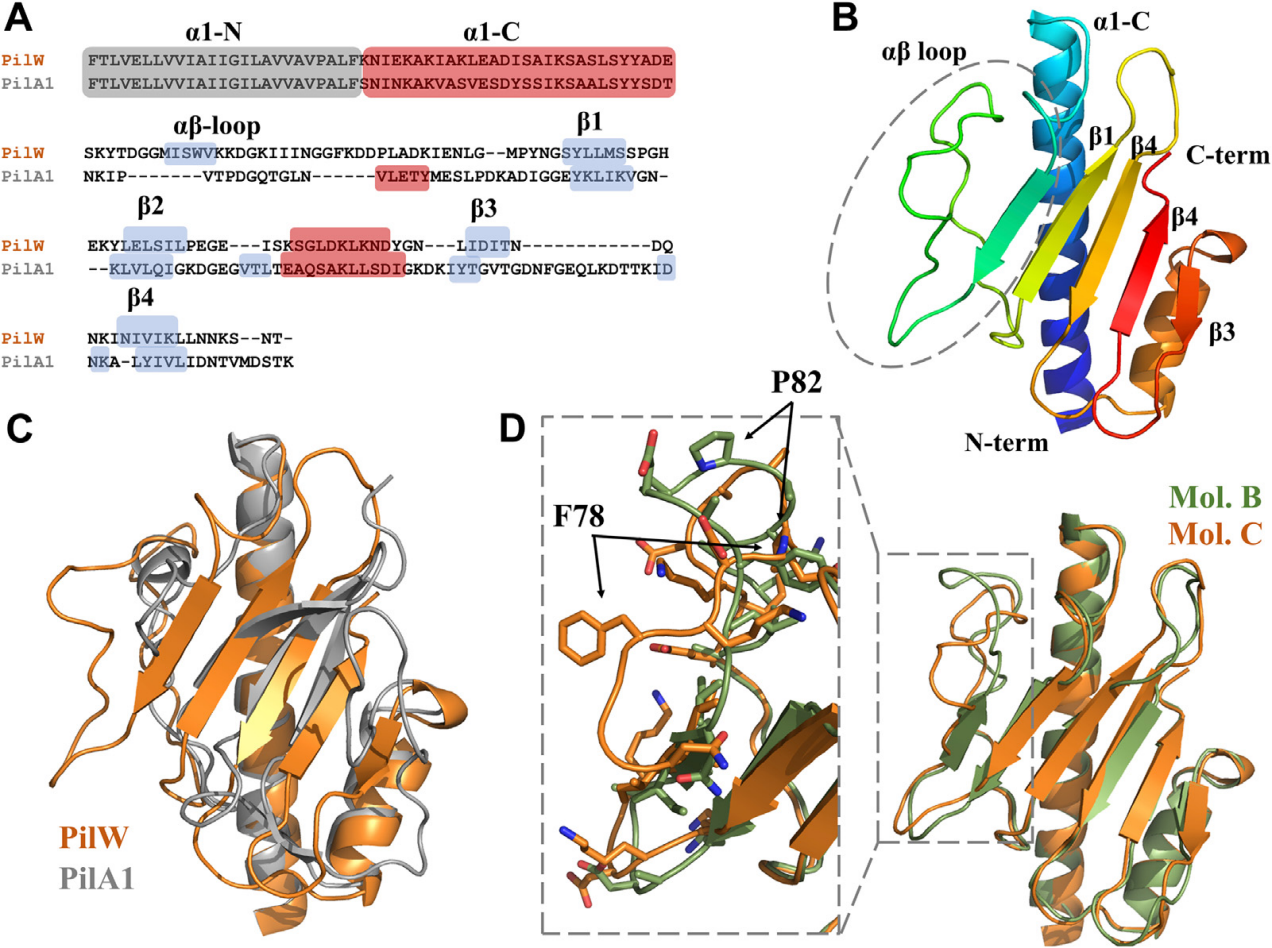
**X-ray crystal structure of PilW.***A*, sequence alignment of PilW and PilA1. *B*, 3D structure of PilW (molecule B), colored in a spectrum from *blue* (N-terminus) to *red* (C-terminus). *C*, superimposition of the PilW (molecule B, *orange*) and PilA1 (*gray*) structures. *D*, detail of the PilW αβ loop, superimposition of molecules B (*olive*) and C (*orange*).

Like PilA1, PilW has a single transmembrane helix (α-1N) followed by a soluble domain and a central α-helix (α-1C) with a β-sheet packed against it. Figure 4*A* shows a sequence alignment of PilW and PilA1 by PROMALS3D (69), highlighting the overlap in predicted secondary structure; the soluble domains (that is excluding the TM helix, residues 1–25) of PilW and PilA1 are 30% identical with the majority of the overlap occurring in the α-1C helix. Figure 4*B* shows the soluble portion of PilW colored from blue (N-terminus) to red (C-terminus) and in Figure 4*C*a superimposition of the PilA1 (gray) and PilW (orange) soluble domains. The majority of the two structures is superimposable, including the four strands of the PilA1 β-sheet and the C-terminal α-helix (which is also found in a superimposition of PilA1 and PilJ); the most obvious point of divergence is the αβ loop, which has a short helical section in PilA1, but adds two strands to the β-sheet of PilW. This loop appears to be highly flexible; in the four molecules of the PilW asymmetric unit, it can be traced in two distinct conformations (chains B and C), with insufficient density to resolve it in the other two. These two molecules differ in the conformations of all of the surface loops despite their general similarity (RMSD: 1.8 A) with the vast majority of the difference stemming from the αβ loop. (Fig. 4*D*).

The comparison of the PilA1 and PilW structures also presents an opportunity to explain the unusual immunogenic cross-reactivity of the proteins. Maldarelli *et al.* found that murine antisera generated from PilW was cross-reactive against all six *C. difficile* T4P proteins tested, PilA1 (CDR20291_3350), PilA2 (CDR20291_3155), PilU (CDR20291_3344), PilV (CDR20291_3345), PilW (CDR20291_2191), and PilJ (CDR20291_0683) (63), implying that some common structural motif(s) exist beyond the conserved N-terminal TM helix. In fact, the greatest response to PilA1, which was generally poorly immunogenic, was from the PilW antisera, and across the six mice immunized with PilA1, responses to PilW were slightly higher than those to PilA1. Our comparison of the two structures reveals a region of striking similarity on what would be the interior face (buried in the assembled pilus), which may help to explain both the antibody cross-reactivity and the incorporation of multiple subunits into T4P fibers (Fig. S5).

Like other type IV pilin proteins from Gram-positive bacteria (26, 50), PilW lacks the C-terminal disulfide bond found in the vast majority of their counterparts from Gram-negative bacteria (29). Unlike PilA1, PilW does not have a two-strand anti-parallel β-sheet in its place, despite the overall similarity of the two structures in this region. However, contrary to our expectations, the Prediction of the Stability Curve of Proteins (SCooP) algorithm (70) predict greater thermal stability for PilW. We verified this prediction using differential scanning calorimetry and CD as shown in Fig. S6; both methods show slightly greater thermostability for PilW. One potential explanation is the amount of buried surface area for each protein which is greater for PilW than PilA1, despite the former’s wider central β-sheet. Calculations of solvent-excluded surface area using a 1.4 Å probe give buried surface area values of 7912 Å^2^ for PilA1 and 8843 Å^2^ for PilW.

The structure of PilW is broadly representative of type IV pilins; the results of a structure-based search of the PilW coordinates using DALI (http://ekhidna2.biocenter.helsinki.fi/dali/) are primarily T4P subunits; the top 10 are all subunits from the structurally related *Streptococcus typhi* pilus (PilS, 3FHU), ETEC longus pilus (CofA, 3S0T), and the *V. cholerae* TCP (TcpA, 3HRV, 1OQV). All of these subunits are from type IVb pilus systems, which we previously noted resemble the *C. difficile* pilus (26). Surprisingly, PilA1 does not appear, even in the top 100 results, but of those 100, 89 are various pilin subunits and 9 are various DNA- or RNA-binding proteins, including a poly(a)-specific ribonuclease (2A1S), which suggested nucleotide binding as a possible mechanism for the defect in biofilm formation.

### Modeling incorporation of PilW and PilJ into *C. difficile* T4P

**Figure 5:**
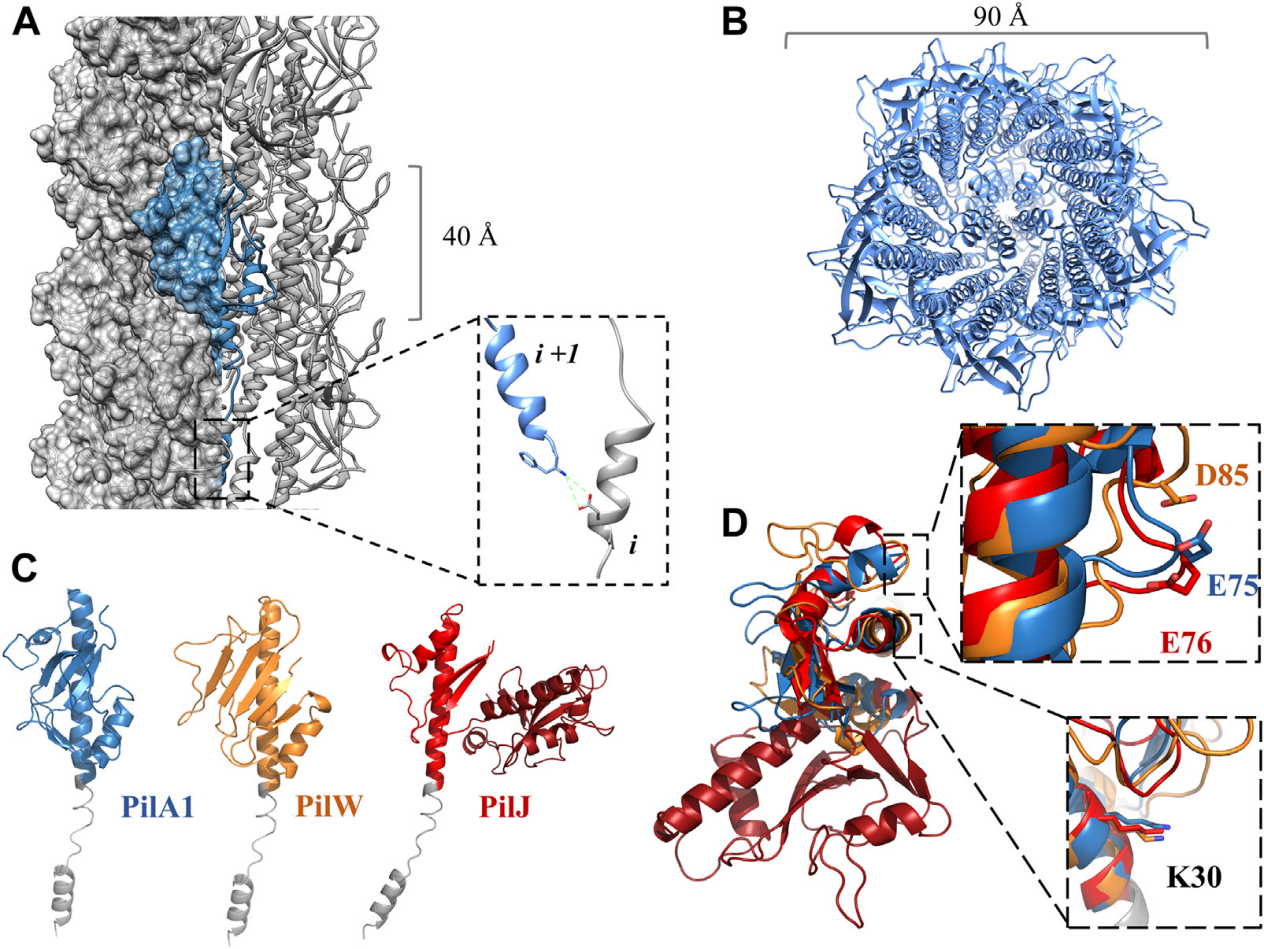
**Incorporation of multiple subunits in *Clostridioides difficile* T4P.***A*, side view of *C. difficile* T4P model based on the ‘melted’ helix found in recent cryo-EM reconstructions; the salt bridge between glutamate 5 and the amino terminus of the next subunit is shown as an inset panel. *B*, *top-down* view of the pilus model. *C*, full-length models of PilA1, PilJ, and PilW with the α1-N (*gray*) based on cryo-EM reconstructions of intact T4P fibers. *D*, superimposition of PilA1 (*blue*), PilJ (*red*), and PilW (*orange*), Lysine 30 and its proposed salt-bridge partners are shown as inset panels. T4P, type IV pilus.

The inclusion of accessory pilins into T4P at low frequency is an obvious mechanism for the incorporation of divergent molecular surfaces. To model the consequences of PilW incorporation, we created an atomistic model of the *C. difficile* T4P fiber. We modeled the N-terminal TM helices using the extended conformation resolved for the near-atomic cryo-EM reconstructions of *P. aeruginosa* and *Neisseria meningitidis* T4P (64) as the conservation of these domains between T4P subunits from different organisms is such that we would expect the extension and ‘melting’ of the helix to be a general feature of T4P and related fibers. Figure 5 shows the model composed entirely of PilA1 in side (Fig. 5*A*) and top-down (Fig. 5*B*) views; the width of the pilus model is ∼9 nm, wider than the type IVa pili for which we have high-resolution cryo-EM reconstructions because of the somewhat bulkier PilA1 headgroups.

Figure 5*C* shows full-length models of PilA1, PilW, and PilJ based on our x-ray crystal structures of the soluble domains. As described above, the a1-N regions (in gray) for these three proteins are similar in sequence and are modeled with identical backbone conformations to model their incorporation into the fiber. Figure 5*D* shows the conserved interaction residues previously identified by our group for assembly of *C. difficile* T4P and by Craig *et al.* in the *V. cholerae* TCP (28), Lysine 30, and Glutamate 75 (PilA1)/Glutamate 76 (PilJ). Equivalent residues in similar positions can be observed in the PilW structure, at Lysine 30 and Aspartate 85. This conservation agrees with the hypothesis that accessory subunits must maintain certain structural motifs for incorporation into T4P fibers even in the absence of general structural similarity.

**Figure 6:**
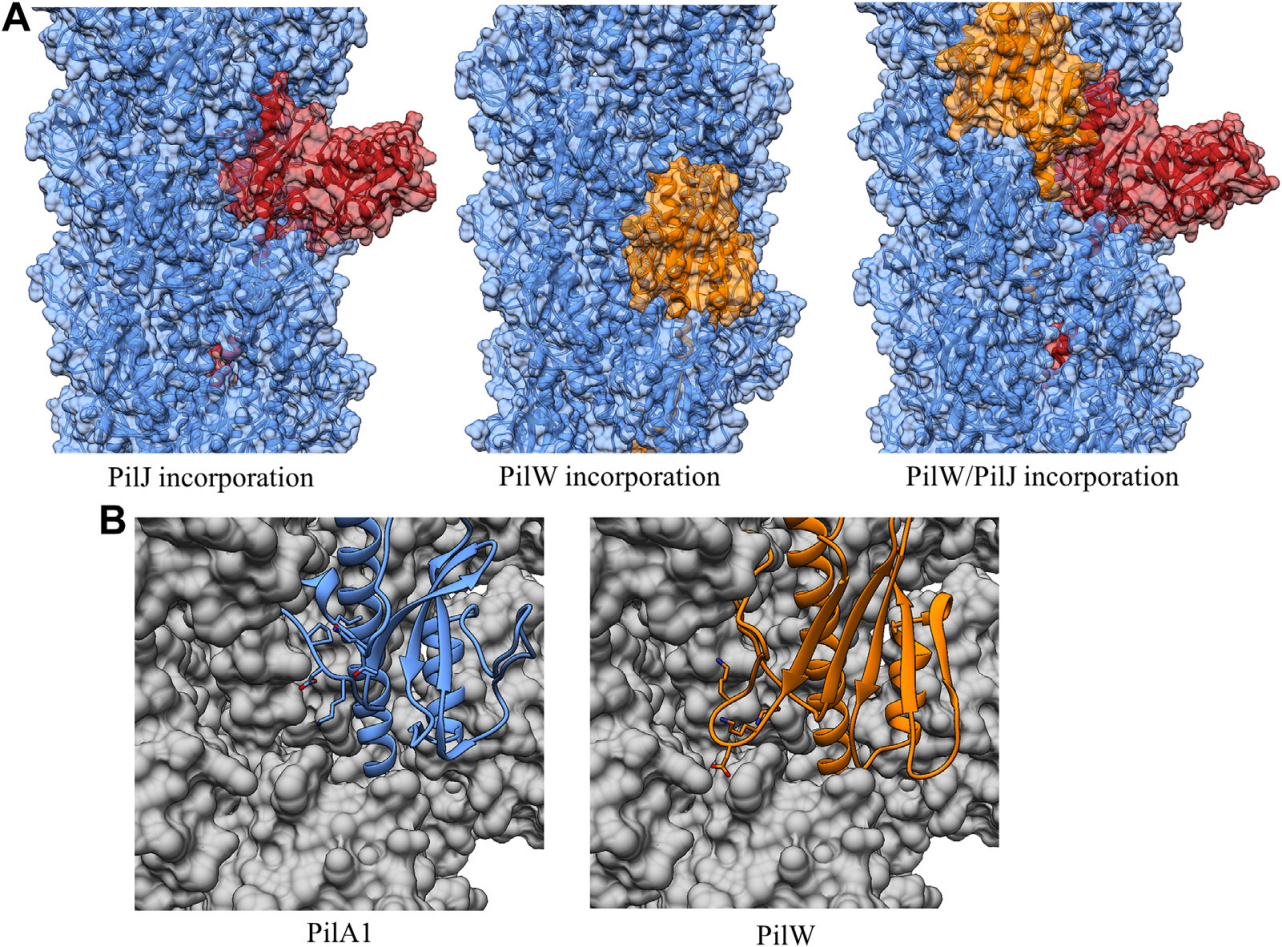
**Heterologous type IV pilus models.***A*, fiber models showing the incorporation of PilJ and PilW into the *Clostridioides difficile* T4P fiber. *B*, groove between adjacent subunits with PilA1 and PilW subunits modeled in. T4P, type IV pilus.

In Figure 6*A*, models of *C. difficile* T4P are shown with and without the incorporation of PilJ and PilW. While the C-terminal domain of PilJ extends out from the pilus fiber, PilW is nearly identical to PilA1 in size (the mature proteins, without the pre-pilin leader sequence, are 185 and 179 amino acids, respectively); but due to its extended αβ loop, PilW is wider along the helical axis of pilin incorporation than PilA1 or PilJ. The conformer found in chain C of our crystal structure can be accommodated in our pilus model with only minor adjustments to the backbone, while the conformer in molecule B would require a total rearrangement, leading us to conclude that the chain C conformation is close to the native conformation found in the assembled fibers. When PilW subunits are modeled in, the αβ-loop of PilW extends into a groove between PilA1 subunits. Figure 6*B* shows PilA1 and PilW subunits in an identical position; the extended conformation of the PilW αβ-loop alters the exposed interaction surface, occluding region of neighboring subunits.

Although both PilJ and PilW can be incorporated into our model individually without significant changes to the conformation of the surrounding PilA1 molecules, our model suggests there is some preference in their relative position if incorporated at adjacent positions. Relative to PilJ, PilW is easily accommodated in the i + 1 position (that the next pilin along a helical axis extending out from the bacterial cell, in reverse order of polymerization). However, in the i-1 position, the αβ loop of PilW would clash with the loop formed by residues 176 to 184 of PilJ (Fig. S7). However, if there is no bias in the polymerization machinery, one would expect this to occur rarely; although PilA1, PilJ, and PilW are the most highly expressed subunits in strain R20291 based on RNA-seq by our group (9) and qPCR by Purcell *et al*. (8), we estimated that there are ∼2000 molecules of PilA1 for each molecule of PilJ (26), and *pilW* is less highly transcribed than *pilJ*.

### PilJ and PilW bind DNA *in vitro*

As described above, *C. difficile* T4P mutants show pronounced defects in *in vitro* biofilm formation. Similar patterns have been observed for T4P mutants in other bacterial systems (21, 40, 71, 72, 73), but because of the diverse array of functions ascribed to T4P, defining the mechanistic basis for these effects remains difficult. Conceptually, we considered four possible mechanisms for the nucleation of biofilm through T4P-dependent effects; (i) the bundling of type IV pili between adjacent bacterial cells, (ii) the adhesion of T4P to bacterial cell-surfaces, (iii) interactions between T4P and polysaccharides in the extracellular matrix, and (iv) recognition of extracellular DNA by T4P (See Fig. S8). Of these, (i) and (iv) are supported by the literature demonstrating interactions between enteropathogenic *Escherichia coli* cells through the bundle-forming pilus (74, 75, 76) and DNA binding by T4P in competent bacteria (77, 78, 79, 80, 81).

While *C. difficile* does not exhibit natural competence *in vitro*, natural transformation has been difficult to detect for many species and many require specifically tailored experimental conditions to observe (46, 82). Extracellular DNA is clearly required for *C. difficile* biofilm stability (Fig. 3*B*). If T4P–DNA interactions contribute to biofilm formation, we would expect to see DNA binding by the recombinant subunits similar to what has been reported previously; DNA-binding T4P subunits have been identified in *N. meningitidis* (78, 83) and *Thermus thermophilus* (81). These proteins, ComP and ComZ respectively, show no sequence or structural similarity; but in both cases, DNA binding could be observed *in vitro* using the soluble domains of individual pilin subunits even in the absence of the identification of a specific DNA uptake sequence.

**Figure 7:**
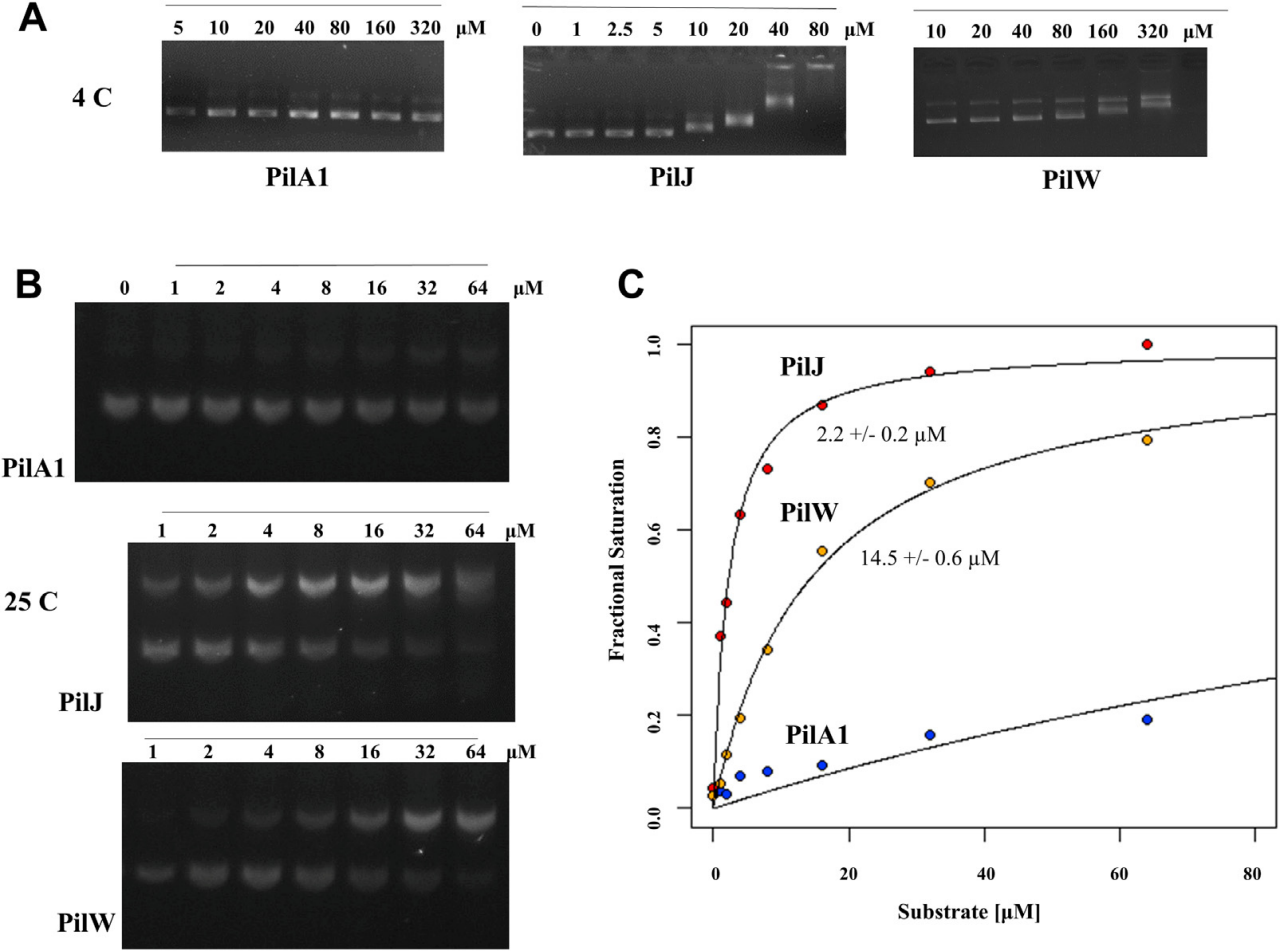
**DNA binding by *Clostridioides difficile* T4P subunits.***A*, EMSA assessment of DNA binding at 4C; *B*, EMSA assessment of binding at 25C. *C*, quantification of affinity for DNA at 4C. T4P, type IV pilus.

Figure 7*A* shows the results of EMSAs for recombinantly expressed soluble PilA1, PilJ, and PilW. PilA1 shows no shift, indicating an absence of any stable protein–DNA complex, while both PilJ and PilW show concentration-dependent shifts in the motility of the DNA, with the reduction in motility indicating DNA binding. In Figure 7*B*, we quantify the binding of PilJ and PilW for plasmid DNA at 25C, where saturation could clearly be observed for both. The apparent affinity constants under these conditions are 2.2 ± 2 μM for PilJ and 14.5 ± 0.6 μM for PilW. Importantly, these values are only of relative importance as they appear to vary considerably based on the temperature, salt concentration, and whether linearized or supercoiled DNA is used.

To understand how PilJ and PilW might recognize DNA, in the absence of a clear DNA-binding motif, we analyzed the surface electrostatics of both proteins and found that while neither had a basic patch equivalent to that of *N. meningitidis* ComP (78), both had basic regions which would be exposed in the assembled pilus based on our model (Fig. 4, *C* and *D*).

### Pangenomic variation in pilW

**Figure 8:**
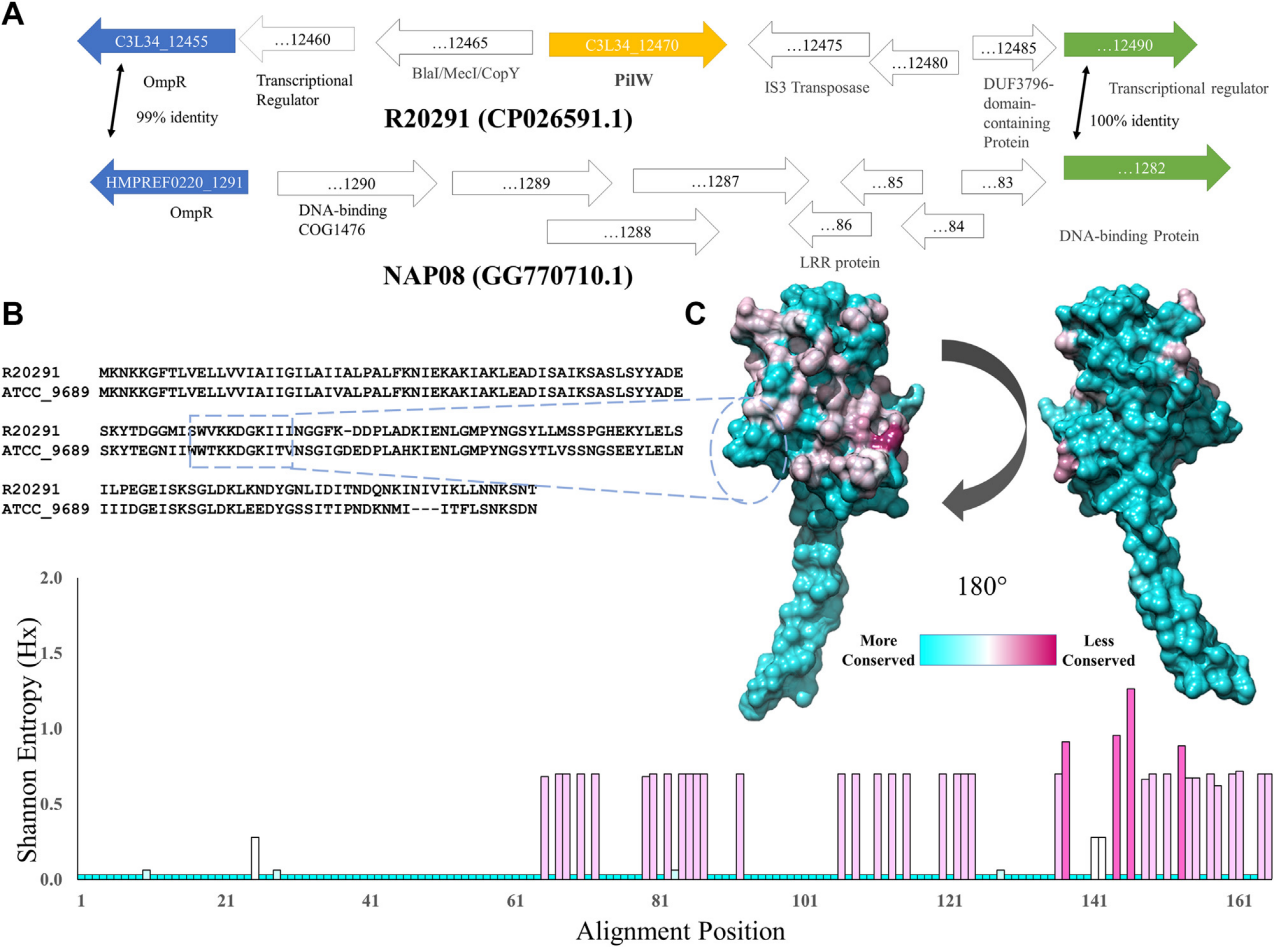
**Pangenomic analysis of *pilW* genes.***A*, analogous regions of the *Clostridioides difficile* R20291 and NAP08 genomes highlighting the presence and absence of *pilW*. *B*, alignment of PilW from R20291 and ATCC. *C*, PilW based on sequence conservation from *cyan* (totally conserved) to *maroon* (highly variable) and Shannon entropy plot from the PilW alignment.

Uniquely among the nine pilin subunit genes which have been identified in *C. difficile*, *pilW* is not present in all strains. Maldarelli *et al.* identified *pilW* genes in 13 of the 19 fully-assembled genomes available at that time (63). Our analysis of 237 *C. difficile* genomes has identified *pilW* genes in 206 genomes. To confirm that these absences are true negatives and not simply the genes too divergent to be identified algorithmically, we compared the region where *pilW* is found universally in the *pilW*+ strains to the equivalent regions in the 31 *pilW* strains. Figure 8*A* shows a comparison of equivalent regions of the R20291 and NAP08 genomes, bounded by two conserved genes, OmpR (C3L34_12455 and HMPREF0220_1291) and an unnamed putative transcription factor (C3L34_12490 and HMPREF0220_1282). Recombination in the region between these two genes results in the appearance of *pilW* in R20291, while no T4P subunits appear in this region of the NAP08 genome. Table S1 lists the identified *pilW* strains, which are heterogeneous and distributed across several ribotypes. We note the appearance of multiple 078 (M120, NAP07, NAP08, T20, QCD23m63), 027 (CIP 107932, QCD37x79, BI1), and 017 (CF5, M68, E13) strains.

Despite its unusual absence in many *C. difficile* strains, when present, PilW is, like PilJ, well conserved; Figure 7*C* shows a Shannon entropy plot for PilW based on our pangenomic alignment. Those residues which vary are clustered on the surface in regions which would be surface exposed on the assembled pilus. Notably, the lysine-rich portion of the αβ loop shown in Figure 4*D* (residues 67–72) is conserved across the alignment (Fig. 7*B*), supporting the hypothesis that this motif is important for DNA recognition.

## Discussion

DNA binding by T4P represents an attractive hypothesis for the mechanism by which they promote biofilm formation in no small part because of the established importance of eDNA in bacterial biofilms. In the *in vitro* biofilm experiments described here and in numerous other studies, the depletion of extracellular DNA retards biofilm formation and can even disperse biofilm at early time points (65, 66). Similarly, defects in the secretion of DNA impede biofilm formation in *Haemophilus influenzae* (84). The polypeptides which interact with eDNA in bacterial biofilms can be conceptually divided into two classes, (i) proteins which are secreted and cross-link eDNA to stabilize the extracellular matrix and (ii) proteins which are bound to cell surface and mediate the attachment of bacterial cells to the extracellular matrix, the latter could be monomeric membrane proteins or incorporated into surface assemblies such as T4P (85).

Indeed, T4P are part of a larger class of helically-assembled extracellular filaments which also includes competence (com) pili and tad/flp pili, under the umbrella of type IV Filaments (T4F or TFF) (86). These fibers are established as essential for natural competence (through DNA uptake) in dozens of species of bacteria across a wide variety of bacterial taxa, Gram-positive and Gram-negative (35). T4F subunits all possess N-terminal TM helices, but their C-terminal headgroups vary considerably, from T4P, which universally have β-sheets packed against the soluble region of the α-helix (30), to competence pili which have three-helix bundles (87) to tad/flp pili which appear to have only short loops following the α-helix (88). Representatives from all three T4F subclasses have been shown to mediate DNA uptake despite the complete lack of identifiable homology between their subunit proteins.

However, while only a few DNA-binding pilus subunits have been identified, some general trends appear to exist within related fibers. DNA binding has been shown to occur at the tips of several related T4P systems from Gram-negative bacteria (type IVa, T4aP) including *V. cholerae*, *N. meningitidis*, and *T. thermophilus* (79, 81, 89). Conversely, DNA binding by competence pili in Gram-positive *Streptococcus pneumoniae* occurs along the length of the pilus, rather than the tip, despite the absence of DNA binding by the major subunit (ComGC) (90, 91), which suggests the incorporation of a DNA-binding subunit sporadically throughout the fiber in a manner similar to *C. difficile* PilJ and *N. meningitidis* PilV and, we presume *C. difficile* PilW. The ability of PilJ to bind to DNA suggests a functional relation between the *C. difficile* T4P system and the *S. pneumoniae* com system rather than the more closely homologous T4Pa systems. We propose that this distinction stems from differences in polymerization/secretion between Gram-positive and Gram-negative T4F systems, which impact the ability of DNA to be taken up depending on whether it is bound at the tip or along the length.

In terms of the specifics of molecular interactions between T4P and DNA, the identification of PilJ and PilW as DNA-binding subunits does little to establish a general pattern because they have little structural similarity to ComP or ComZ; much less than the major pilin subunits of these three T4P systems, PilA1 (*C. difficile*), PilA4 (*T. thermophilus*), and PilE (*N. meningitidis*). The implication of this diversity in DNA-binding T4P subunits, both in their manner of incorporation (specific incorporation at the tip *versus* sporadic incorporation along the length of the fiber) and their molecular structures implies that DNA binding evolved separately in these species after T4P were established. Correspondingly, we may hypothesize that the ancestral T4F system did not bind DNA and that the evolution of natural competence began with the addition of DNA binding to T4F fibers already capable of retraction for surface motility.

However, despite the wide variety of minor pilin structures, including DNA-binding subunits, within a given species, minor pilin subunits appear to be universally more conserved than the major subunit (24, 40, 92, 93). That is, the pattern we observe for PilJ, PilW, and PilA1 is replicated in other T4P system; the major subunit has the least conserved amino acid sequence within a given taxonomic group. For commensal and pathogenic bacteria, this pattern can be explained in terms of diversifying selection pressure on the major subunit because of its greater abundance. In this context, adhesive interactions would have to occur through minor subunits, either as tip adhesins (GspK, CofB, ComZ) or through the addition of minor pilin subunits incorporated sporadically; either would allow for a conserved interaction surface at relatively low abundance.

Because *pilJ* and *pilW* are (uniquely among the nine *C. difficile* T4P subunits) found outside of a cluster of T4P-related genes, we have hypothesized that they are later additions to the T4P system; accessories rather than core components. For *pilW*, this is doubly probable because it is not found in all *C. difficile* genomes, but its pattern of presence and absence is not correlated with overall taxonomy. Pangenomic analysis of *pilW* genes indicates that *pilW* is present across a wide range of *C. difficile* strains and *pilW* strains can be found in multiple, seemingly unrelated taxa (ribotypes 027, 017, 078). These features suggest to us that the *C. difficile* common ancestor was *pilW*+ and those strains which lack *pilW* have lost it through recombination. However, no *pilW* homolog can be detected in the genome of *Paeniclostridium sordellii*, in which other T4P genes, including *pilA1* and *pilJ*, are well conserved with their counterparts in *C. difficile*.

One important outstanding question is, given that fairly divergent structures can be incorporated into T4P fibers, why are accessory subunits like PilW found only in some strains within a species, not more common? A key factor in their occurrence may be sporadic incorporation, as subunits at the pilus tip are the first to be incorporated into the developing fiber (the reverse of flagellar synthesis) and the importance of that initiation may act as a barrier to diversification. While many minor pilins which form part of a tip initiation complex can be identified by homology to the tip complex of the type II secretion pseudopilus (GspH, GspI, GspJ, and GspK), it is unclear for many other minor pilins whether they are incorporated as solely at initiation or sporadically. Experimentally, this can be difficult to determine; for immunogold TEM, even small amounts of cross-reactivity with the major subunit could provide false positive results for sporadic incorporation. Relative abundance could be a useful indicator as sporadic incorporation should require greater abundance than incorporation solely at the tip, just as the major pilin can be identified by its greater abundance. However, while our previous quantification of transcription levels by RNA-seq did show *pilA1*, *pilJ*, and *pilW* as the most abundant transcripts in strain R20291, Purcell *et al.*’s comparison of transcription levels in R20291 and 630 showed much lower transcription levels in strain 630 for *pilA1* and *pilW* and much greater transcription for *pilA5* (8). Similarly, divergent results can be found for changes in the expression of T4P subunits during biofilm formation; Maldarelli *et al.* found a small but significant downregulation in the expression of *pilW* in strain R20291 during biofilm formation (9), whereas Tremblay *et al.* found *pilW* to be upregulated in strain 630 (58).

In short, the ability of minor T4P subunits to functionalize pilus fibers with DNA-binding subunits has a measurable impact on biofilm formation in *C. difficile*, suggesting that the evolution of DNA binding by T4P has not been driven solely by DNA uptake. DNA binding by T4P occurs through diverse molecular mechanisms, less conserved than the overall pilus architecture. We anticipate that as more DNA-binding subunits are identified and their structures resolved, we will see a wide variety of modes of DNA recognition, some optimized for recognition of specific DNA sequences and others with little or no sequence specificity. With this diversity of structure comes a diversity of function as DNA binding by T4P systems may impact a range of functions beyond DNA uptake in bacterial species with and without demonstrable natural competence.

## Experimental procedures

### Bacterial strains and growth conditions

The *C. difficile* R20291 strain was used for these experiments as its genetic sequence is known and mutants of the T4P system are available. Generation of the R20291 *pilA1::ermB*, R20291 *pilJ::ermB*, R20291 *pilW::ermB*, and R20291 *pilA1::ermB* ppilA1 *C. difficile* strains were previously described (Piepenbrink *et al.* 2015); the ppilA1 complement was made by cloning pilA1 into the p84151 modular plasmid using the NotI and XhoI restriction enzymes. Gene interruptions were confirmed by colony PCR using the following forward and reverse primers: *pilA1F* (5′ cccaaattatctgctgtaacacttgta), *pilA1R* (5′ gcagtagtggcagttccagctttattt), *pilJF* (5′ ggcagttacatgtctttctaatagag), *pilJR* (5′ ggaccccatccctctttagaatg), *pilWF* (5′ ggcaataatagcacttccagc), *pilWR* (5′ gaccacttttgcttatttctcc). Insertion of the compliment was confirmed by colony PCR using the forward primer (*thio-F1*, 5′ ctactagtacgcgttatattgataaaaataataatagtgg) and reverse primer (*ermB-R1*, 5′ gcgactcatagaattatttcctccc). Strains were maintained on BHISTA plates (37 g/l Bacto brain heart infusion, 5 g/l yeast extract, 10% w/v L-cysteine, 10% w/v taurocholate, and 1% w/v agar) with antibiotics for gene-interruption mutants or plasmid maintenance as necessary. Overnight cultures of *C. difficile* were a cell scrap from the BHISTA plates grown in 40 ml of BHIS broth (37 g/l Bacto brain heart infusion, 5 g/l yeast extract, 10% w/v L-cysteine) in an anaerobic chamber (Coy Lab Products) with an atmosphere of 5% H_2_, 5% CO_2_, and 90% N_2_. For experiments, *C. difficile* was diluted 1:4 in BHIS for growth and maintenance unless otherwise specified. Antibiotics were used individually for maintaining the gene-interruption mutants at the following concentrations: erythromycin (2.5 μg/ml), cefotaxime (60 μg/ml), and lincomycin (20 μg/ml). Thiamphenicol (15 μg/ml) was used to maintain the compliment plasmid in all experiments.

### Preparation of biofilm for microscopy

Overnight *C. difficile* strain growths were diluted 1:4 in 20 ml of BHIS with thiamphenicol for plasmid maintenance in 10-cm^2^ flat tissue culture tubes (TPP Techno Plastic Products AG) containing upright 1/8 × 1-inch untreated stainless-steel fender washers (Everbilt). Stainless-steel washers were sterilized by washing and autoclaving prior to use. Cultures were grown statically at 37C for 7 days, changing the media by decanting every 24 h. On the seventh day, the media were changed 4 h before pre-stainless steel washer removal to ensure robust germinating growth on the surface of the biofilm. After 4 h, the stainless-steel washers were removed and placed in 6-well tissue culture plates that contained 10% w/v formalin in PBS and incubated for 15 min. After 15 min, the 6-well tissue culture plates were removed from the anaerobic chamber and the rest of the protocol was performed aerobically. The stainless-steel washers were washed with PBS at least 3 times. The stainless-steel washers were then incubated in (1 μg/ml) FM 1 to 43 (Thermo Scientific) for 30 min at room temperature wrapped in foil. Once stained, the steel washed were transferred to PBS and washed twice then mounted using (mounting medium name and company) between a slide (25 × 75 × 1.0 mm) and a coverslip (22 × 22 mm No 1.5). The mounting medium was allowed to polymerize for 48 h at room temperature and then were stored at -20C until imaging as described below.

### Confocal laser scanning microscopy

Biofilms were grown on stainless-steel surfaces and prepared as described above. Imaging was performed using CLSM on a Nikon A1 Confocal System with an upright Nikon Ni-E fluorescent scope and Nikon NIS-Elements. We used the 488 laser line with a PlanApo 60XA/1.2 WI 0.15 to 0.18 WD water immersion lens. Z-stacks were acquired for each of the five stainless-steel washers for each strain with three Z-stacks from each washer. The biomass volume to surface area ratio was analyzed using the Comstat2 software package (http://www.comstat.dk/). The 3D representations of the biofilms were generated using the 3D viewer plugin in FIJI distribution of ImageJ (http://3dviewer.neurofly.de/).

### Preparation of biofilm for crystal violet

Overnight *C. difficile* strain growths were diluted 1:5 in 5 ml BHIS with thiamphenicol for plasmid maintenance in 6-well tissue culture plates. Cultures were grown statically for 7 days at 37C, changing the media *via* pipette every 24 h. On the seventh day, media was removed, and samples were left to dry for 4 to 6 h until ready for crystal violet staining as described below.

### Crystal violet biofilm assay

Dried samples were prepared as described above and then incubated in 1% w/v crystal violet for 10 min, then washed three times with PBS. Samples were then incubated overnight at 4C in 1 ml of 100% ethanol and quantified at the absorbance, corresponding to crystal violet, which was measured at a _λ_570 nm with a nanodrop spectrophotometer (Thermo Scientific NanoDrop One). 100% ethanol was used as a blank. Every experiment had five replicates of each strain.

### DNAse treatment of mature biofilm

Overnight *C. difficile* WT growth was diluted 1:5 in 5 ml BHIS in 6-well tissue culture plates. Cultures were grown statically for 3 days at 37C, changing the media *via* decanting and pipette every 24 h. On the third day, media was removed, and samples were then left to air dry, the media was removed, or the media was changed with to either BHIS or BHIS + 100μg/ml recombinant DNAse (Sigma) (67). After 15 min of exposure to DNAse, the media was removed, and all samples were left to air dry for 4 to 6 h until ready for crystal violet staining as described above.

### Protein expression and purification

For DNA-binding assays, PilA1, PilJ, and PilW (beginning with the first residue of the soluble domain, residue 25 of PilW, 26 of PilA and PilJ from the mature protein) were expressed in pET30a as described previously (26, 94), with the exception that NiCo21 cells were used rather than BL21. Briefly, 300 ml of LB-kan (50 μg/ml) was inoculated from glycerol stocks and grown to saturation overnight. The following morning, 30 ml of this culture was added to six flasks containing 1l of LB-kan and the cultures were grown to an A_600_ of approximately 0.5 at 37C. The temperature was then reduced to 18C and IPTG was added to 500 μM. These flasks were grown for a further 18 h before being harvested by centrifugation at 7000*g* for 20 min. Cells were freeze thawed, resuspended in 20 mM Tris–HCl, 500 mM sucrose, 5 mM NaEDTA, 2 mM MgCl_2_, 50 mM NaCl, and 0.1% NaN_3_ and lysed using lysozyme (0.5 mg/ml), DNAse I (0.02 mg/ml), 1% NaDeoxycholate, and 0.5% triton-X100. The resulting lysate centrifuged again, this time at 20,000*g* for 30 min. The supernatant was purified using a nickel-NTA column on a GE Åkta Start. The Ni elution was further purified through size-exclusion chromatography over a GE S200 Superdex column using an Biorad NGC FLPC. For X-ray crystallography, PilW was cloned into an MBP-fusion vector, making use of previously described surface entropy reduction mutations and a rigid linker terminated with three alanine residues (pMal E) (95), starting with residue 26 of the mature protein (with the addition of a C-terminal 6xHis tag), expressed and purified as described previously (26, 39, 40) again with NiCo21 cells being used for expression.

### X-ray crystallography

MBP-PilW was concentrated to 30 mg/ml (with and without the addition of maltose) in 10 mM Tris–Hcl pH 8.5, 100 mM NaCl and screened for crystallization conditions by sitting drop vapor diffusion using an ARI Gryphon at room temperature. Crystals were observed after 3 days in several related conditions in the Hampton Index screen (82–85, 0.2 M MgCl_2_ hexahydrate, 25% PEG3350, various buffers at 0.1 M) without the addition of maltose. After optimization, the final crystallization conditions were 0.1 M Hepes pH 7.5, 0.2 M CsCl_2_, 25% PEG3350, 0.005% n-Octyl-β-D-glucoside, 0.348 M NaCl, 0.7% ethanol, and 18 mg/ml protein at 4C. Crystals were cryo-protected in mother liquor with 20% ethylene glycol before flash-freezing. Data were collected at Stanford Synchrotron Radiation Labs (SSRL) beamline 12-2 and GM/CA at the Advanced Photon Source, Argonne National Labs, with the final dataset being collected on beamline 23-ID-D. Multiple datasets were collected with either multiple lattices or high mosaicity and anisotropic diffraction, complicating integration and phasing. Ultimately, the addition of NaCl and ethanol increased the proportion of single crystals and growth at 4C and reduced the mosaicity of the final dataset collected on 23-ID-D. With this dataset, we were able to determine that the correct space group was P1 (triclinic) rather than monoclinic.

Reflections were integrated by XDS (96), and after two datasets, from the same crystal were combined in Blend (97), scaled, and merged by Aimless (98) and truncated by STARANISO (99) to account for the anisotropic diffraction. Initial phases were generated by molecular replacement using Phaser (100) using a sequential search of 1) four copies of unliganded MBPand 2) four copies of a truncated PilA1 with all loops deleted. Phenix and Coot were used for generating the initial solution, iterative building, and refinement (101, 102, 103, 104). After numerous cycles of building and refinement (using both refmac and phenix.refine) and validation using Molprobity (105), the resulting structure contains four copies of MBP-PilW, one of which contains a gaps in the αβ-loop and three of which are complete, resulting in a final R-work of 0.186 and R-free of 0.218. The crystallographic parameters of the refined data are summarized in Table 1; geometric and residual statistics were calculated using phenix.Table 1 and Aimless. To reduce the effect of bias on the calculated electron density maps, phenix.maps was used to calculate a composite omit map as well as a feature-enhanced map (FEM) (106) shown in Fig. S4.

### Pilus modeling

Modeling of pilus fibers incorporating PilA1, PilJ, and PilW was performed as described previously (26). Models of full-length PilA1, PilJ, and PilW were created from the crystal structures of the soluble domains using the high-resolution cryo-EM model of the *P. aeruginosa* T4P, which shows a conformation of the a1-N helix expected to be general across T4P systems (64). Based on the similarity of PilA1 and TcpA, an initial model incorporating only PilA1 was created through superimposition using the TCP cryo-EM model (28). Modeling the incorporation of PilJ and PilW individually required replacement of a PilA1 subunit as well as rigid-body minimization using MMTK with AMBER OPLS forcefield with the atoms of the PilA1 subunits fixed. As noted above, the creation of a model incorporating PilJ and PilW in adjacent positions was only possible with PilW in i + 1 position (relative to PilJ).

### Electrophoretic mobility shift assays

*C. difficile* T4P subunits were expressed and purified as described above. For EMSA assessment at 25C, PilA1, PilJ, and PilW were individually incubated for 30 min with 10 ng of DNA in a total volume of 10 μl with 10 mM Tris–HCl pH 8.5, 50 mM NaCl, and 1 mg/ml bovine serum albumin. DNA was separated on a 1.5% agarose gel inoculated with ethidium bromide (EtBr) for 30 min at 50 V. For EMSA assessment at 4C, PilA1, PilJ, and PilW were individually incubated for 30 min with 500 ng of DNA in a total volume of 10 μl with 20 mM Tris–HCl pH 8.3 and 100 mM NaCl. DNA was separated on a 0.8% agarose gel inoculated with EtBr for 45 min at 100V. DNA mobility as a function of protein concentration was measured by UV-visualization of EtBr.

### Twitching motility assay

BHIS+1% glucose and 1.8% agar plates were prepared fresh and allowed to equilibrate in the anaerobic chamber for 24 h prior to use. Thiamphenicol was used for plasmid maintenance. Plates were made by pipetting 8 ml of medium into a 60-mm Petri dish and allowing to air dry with the lid on the sterilized bench. *C. difficile* strains were grown overnight on BHISTA 1% agar plates from a spore stock. After equilibration, each plate was stab inoculated with one colony from their respective overnight strain plate. Plates were immediately placed into a sealable container and grown in the anaerobic chamber for 72 h at 37C. To visualize the bacteria movement, plates were removed from the anaerobic chamber and their agar was also removed. The plates were not washed in between agar removal and staining. The plates were stained using 1% crystal violet, 1% formaldehyde, 1% methanol in PBS for 5 min. After 5 min, the stain was removed, and the remaining liquid was wicked off using kim wipes. The plates were then allowed to air dry for 24 h with the lid off inverted on kim wipes. Once dry, the motility area was quantified by using mm measurements on a ruler, taking measurements of the widest point and a perpendicular measurement. The equation used to find the area was as follows: $\text{Surface Area }=\;\Pi \text{∗}\left(\frac{\text{L}}{2}\right)\text{∗}\left(\frac{\text{W}}{2}\right)$. The experiment was performed with n = 5 per strain.

## Data availability

The structure of MBP-PilW is deposited in the Protein Data Bank under PDB ID 8DX4.

## Supporting information

This article contains supporting information (http://www.pymol.org/pymol).

## Conflict of interest

The authors declare that they have no known competing financial interests or personal relationships that could have appeared to influence the work reported in this article.
