# Supplementary material for: Recognition of extracellular DNA by type IV pili promotes biofilm formation by Clostridioides difficile
Source: J Biol Chem. 2022 Sep 3;298(10):102449. doi: 10.1016/j.jbc.2022.102449 (PMC9556784; doi:10.1016/j.jbc.2022.102449)
Supplement: Supporting Information [file mmc1.pdf]

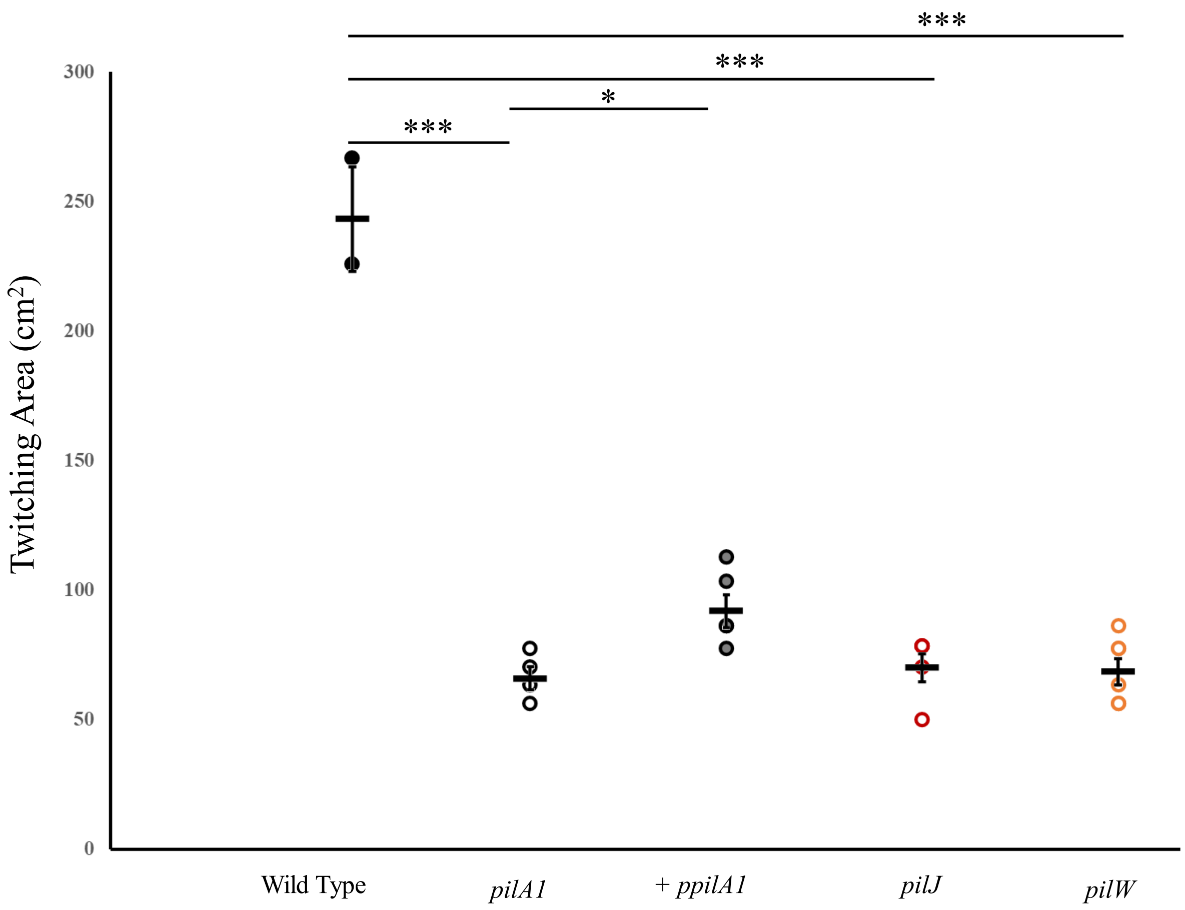

Supporting Figure 1: Twitching Motility by *C. difficile* mutants. Twitching area (average width x average length) for C. difficile R20291 wild type and mutants; bacteria were grown at the interface of 1.8% BHIS agar plates (+1% glucose) and the petri dish for three days. Twitching was assessed by staining with 1% crystal violet + 1% formalin to fix the bacteria to the petri dish.

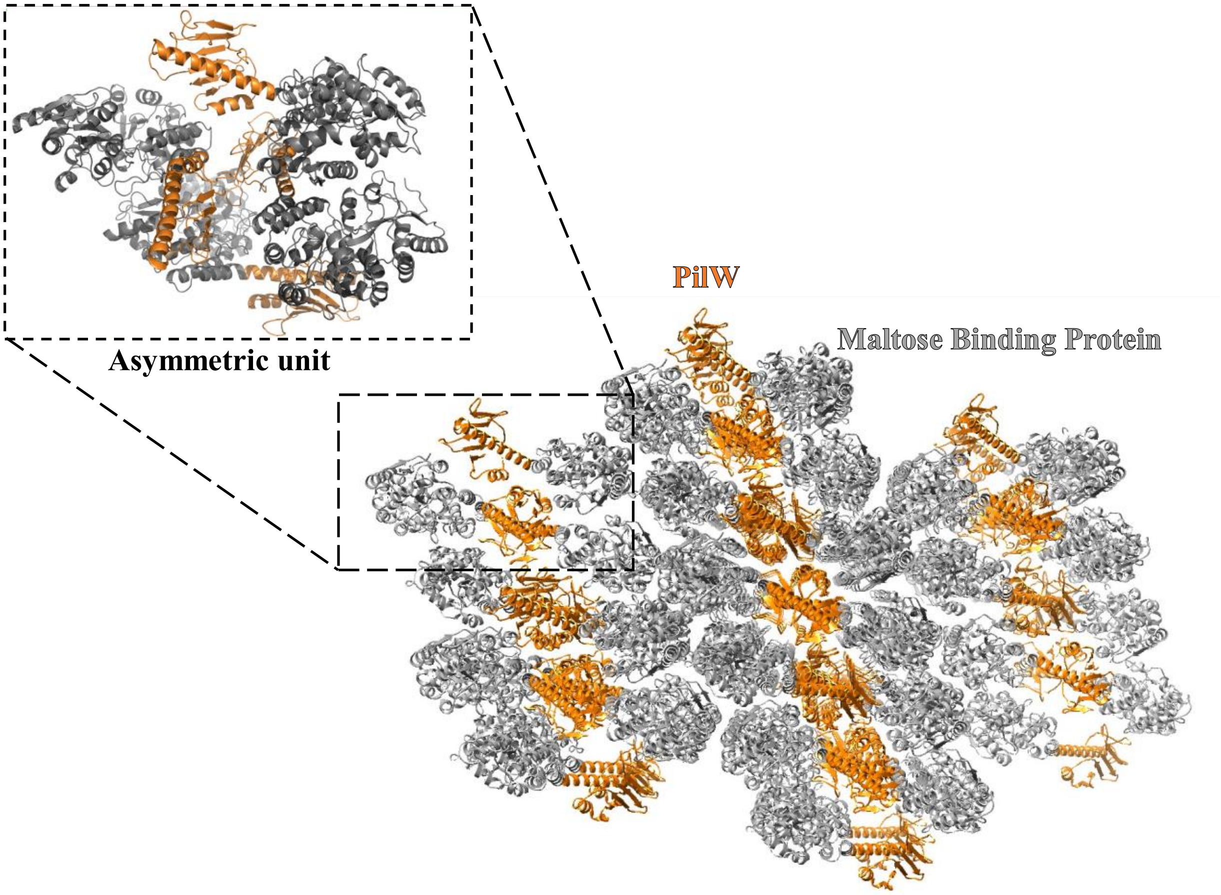

Supporting Figure 2: MPB-PilW crystal lattice. The MBP-PilW crystal lattice is depicted here, with the four molecules of the asymmetric unit shown in the inset panel. The N-terminal Maltose-binding protein (MBP) is in grey and the C-terminal PilW in orange. Images were generated using Pymol.

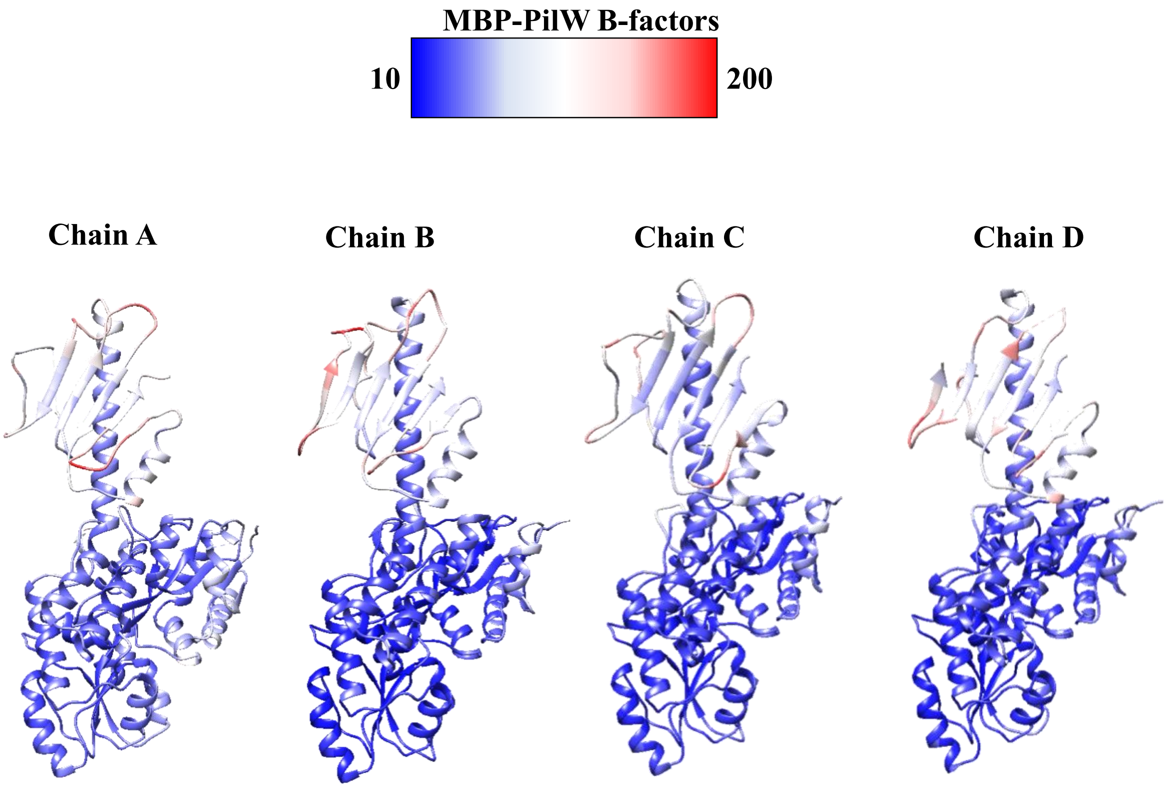
MBP-PilW B-factors
10
200
Chain A
Chain B
Chain C
Chain D

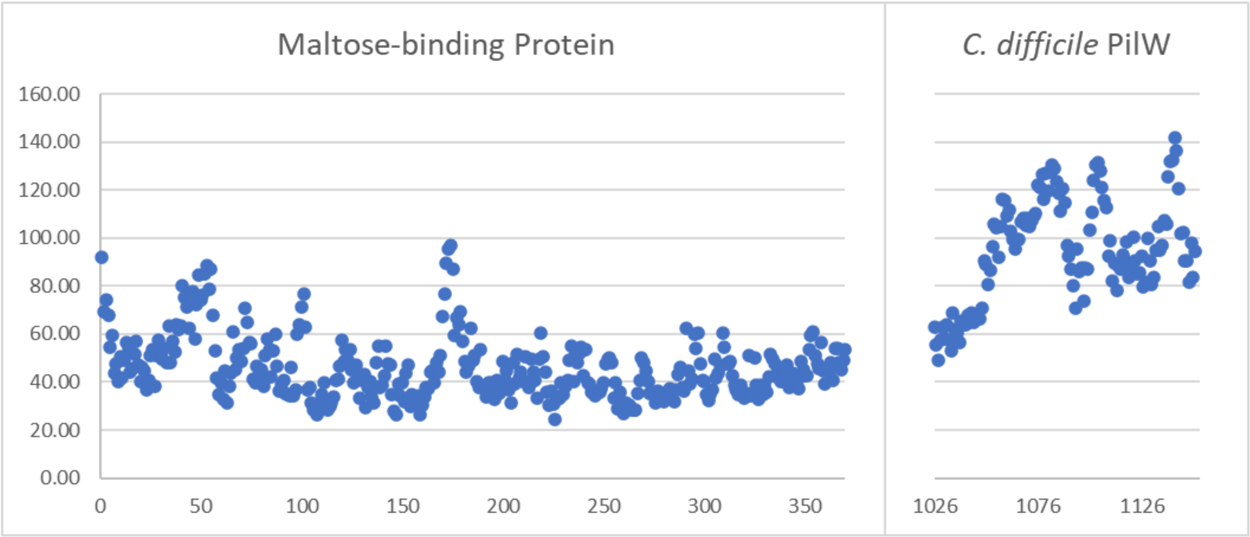
Maltose-binding Protein
C. difficile PilW
160.00
140.00
120.00
100.00
80.00
60.00
40.00
20.00
0.00
0
50
100
150
200
250
300
350
1026
1076
1126

B factors by chain:

MBP (residues 0-370)

A	63.2112043153
B	43.7671838153
C	45.8069082842
D	43.9478256541

PilW (residues 1026-1152)

A	102.054162246
B	107.022144354
C	95.0819262168
D	101.935930551

Supporting Figure 3: Relative B-factors in the MBP-PilW crystal structure. The relative thermal factors (B-factors) for each chain of the MBP-PilW crystal structure are depicted here, ranging from blue (lowest) to red (highest). The lower panel shows the average B-factor by residue across chain C with the average B-factors for MBP and PilW for each chain listed below.

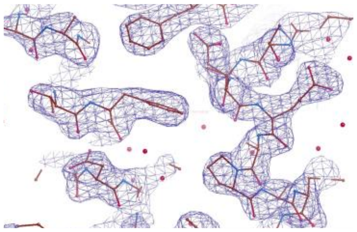

MBP (F194, F250)

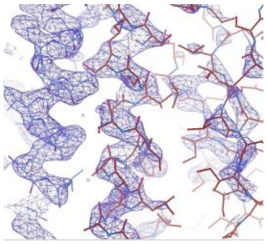

α1-C Helix (26-50)

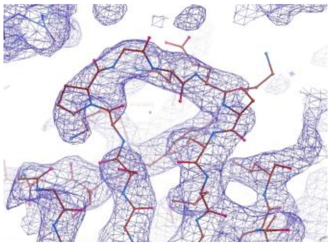

β1-β2 loop (102-110)

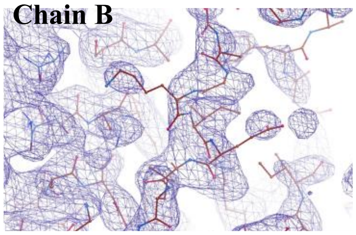

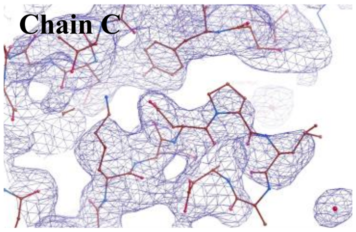

αβ loop (88-98)

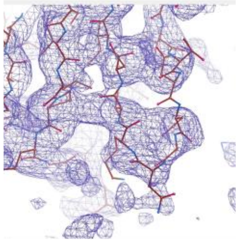

β4-β3 loop (140-145)

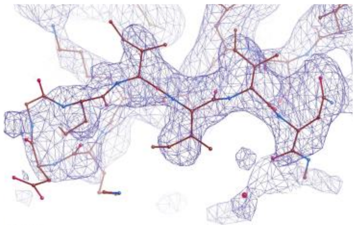

αβ loop (67-73)

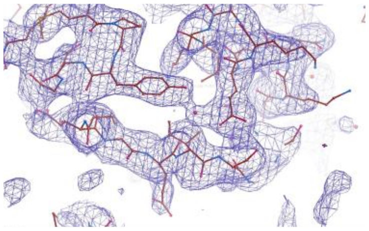

β2 – α2 loop (116-121)

Composite Omit Map

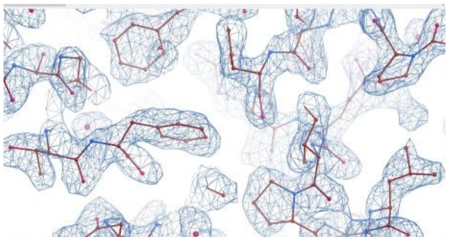

MBP (F194, F250)

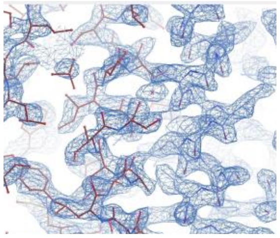

α1-C Helix (26-50)

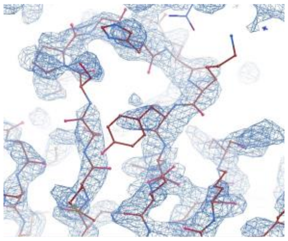

β1-β2 loop (102-110)

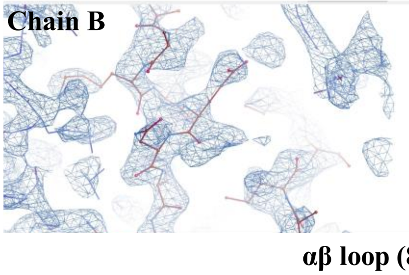

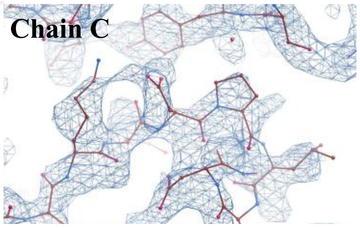

αβ loop (88-98)

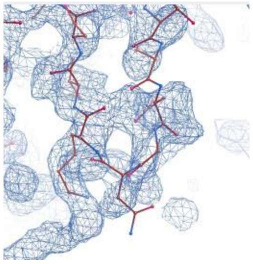

β4-β3 loop (140-145)

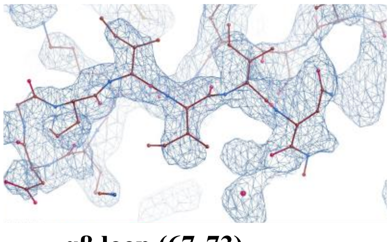

αβ loop (67-73)

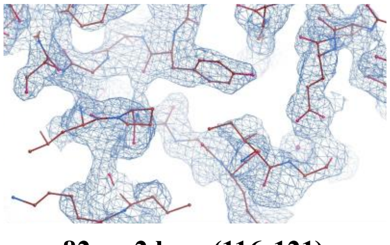

β2 – α2 loop (116-121)

Feature-enhanced map

Supporting Figure 4: MBP and PilW Electron Density and B-factors. Calculated composite omit map (simple, NCS-averaged) and feature-enhanced map of the final MBP-PilW structure are shown here; contoured to 1s and colored purple (composite omit map) and blue (feature-enhanced map).

Supporting Table 1: ***pilW- C. difficile*** **genomes**

Strain	Ribotype
BI1	027
BI9	001
CD002	002
CD175	
CF5	017
CIP 107932	027
DA00065	
E1	126
E10	033
E13	017
M120	078
M68	017
NAP07	078
NAP08	078
P71	
P74	
QCD63q42	001
QCD76w55	027
QCD37x79	027
QCD97b34	027
QCD66c26	027
QCD32g58	027
QCD23m63	078
T20	078
6466	
6503	
6534	
ATCC 43255 (VPI 10463)	087
2007855	
050-P50-2011	
002-P50-2011	

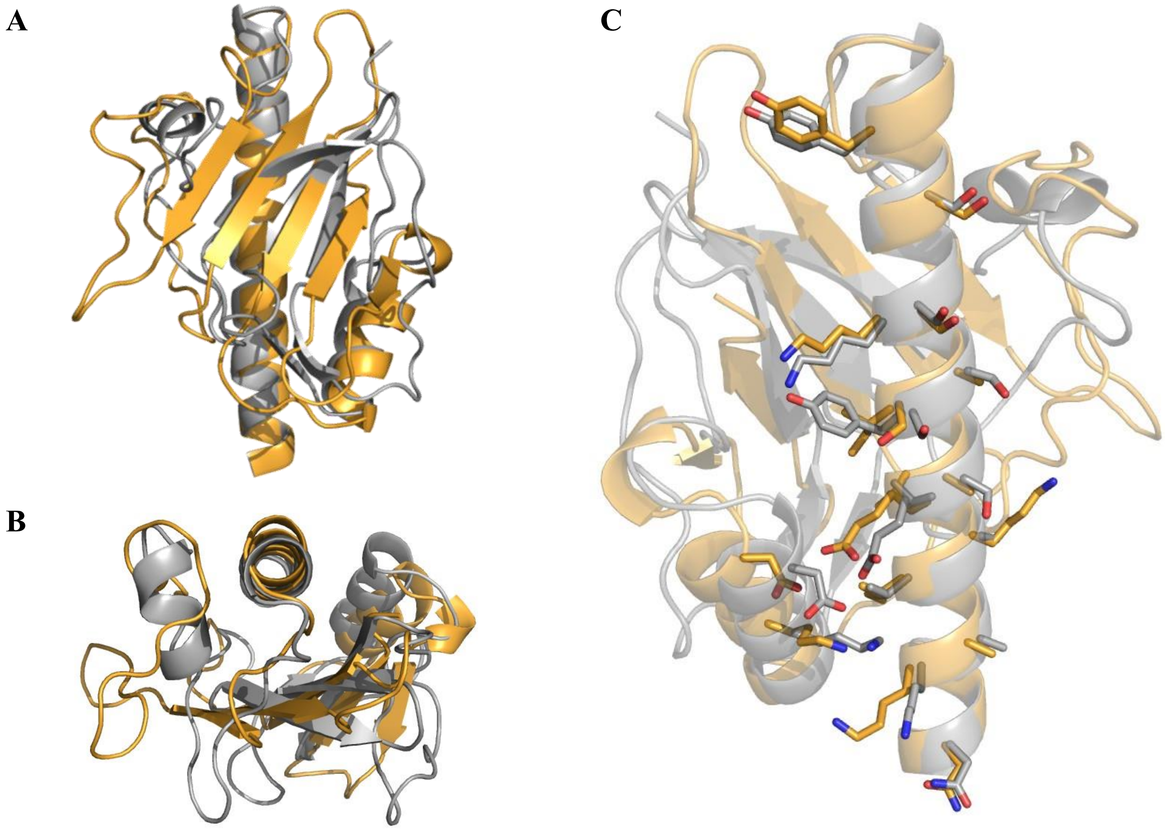

Supporting Figure 5: Conservation between PilW and PilA1. PilW (orange) and PilA1 (grey) are superimposed. Panels A and B show horizontal and vertical cartoon representations, showing the greater structural similarity on the 'back' side, which would face into the pilus fiber. Panel C includes atomistic depictions of residues in the α1-C and C-terminal helices. Images were generated using Pymol.

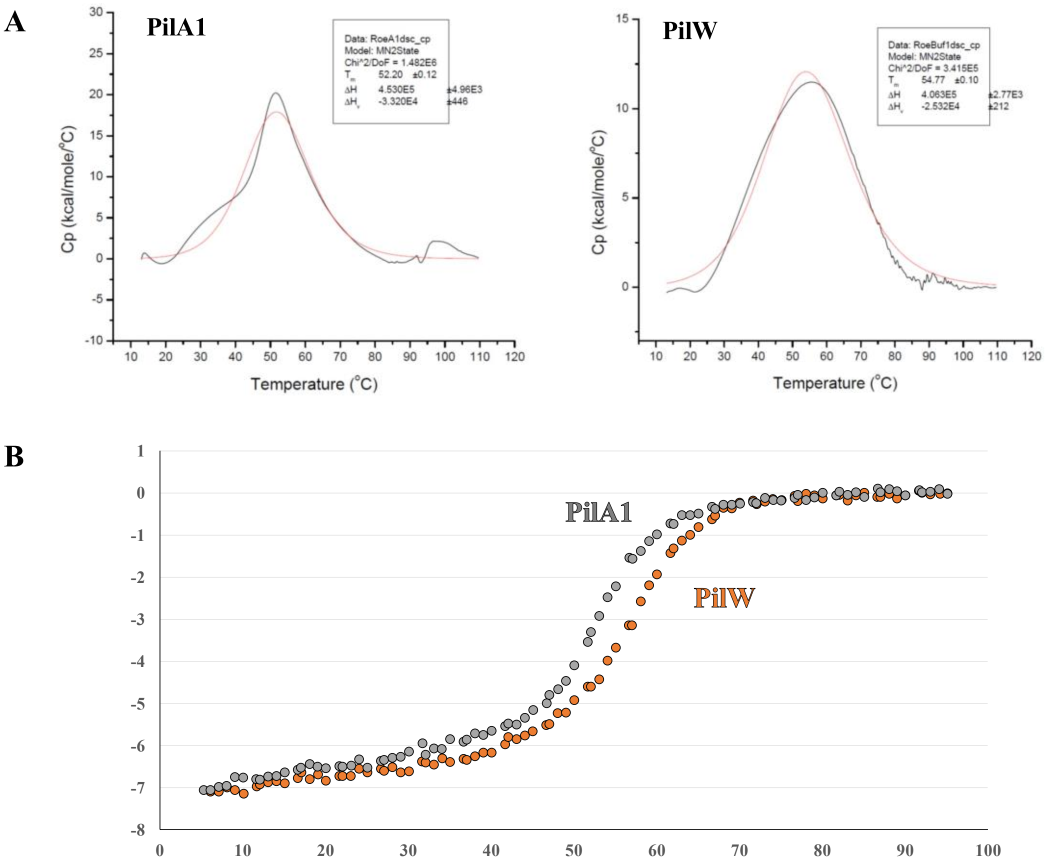

Supporting Figure 6: Stability of PilW and PilA1. Thermal stability of PilW and PilA1 is measured here by differential scanning calorimetry (DSC) in panel A and in circular dichroism (CD) in panel B. Samples at 10 µM were in 20mM Tris-Hcl pH 8.5, 100mM NaCl. DSC data was collected using a MicroCal DSC (Malvern Panalytical) and analyzed using Origin; CD data was collected using a Jasco J-815 spectrophotomer.

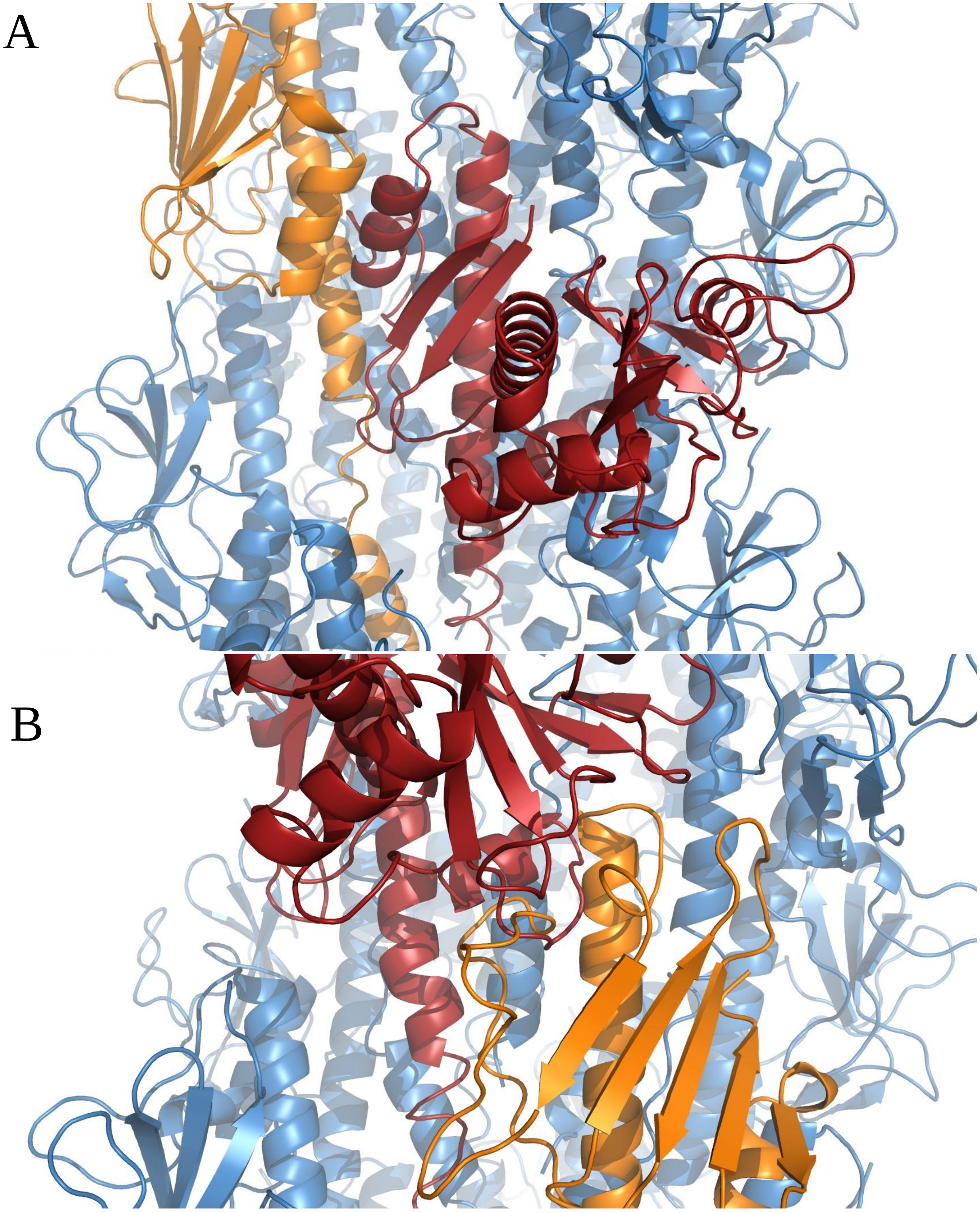

Supporting Figure 7: Incorporation of PilJ and PilW into C. difficile T4P. Panel A shows PilW incorporated at i+1 relative to PilJ (i). Panel B shows PilW incorporated at i-1 relative to PilJ (i). Images were generated using Pymol.

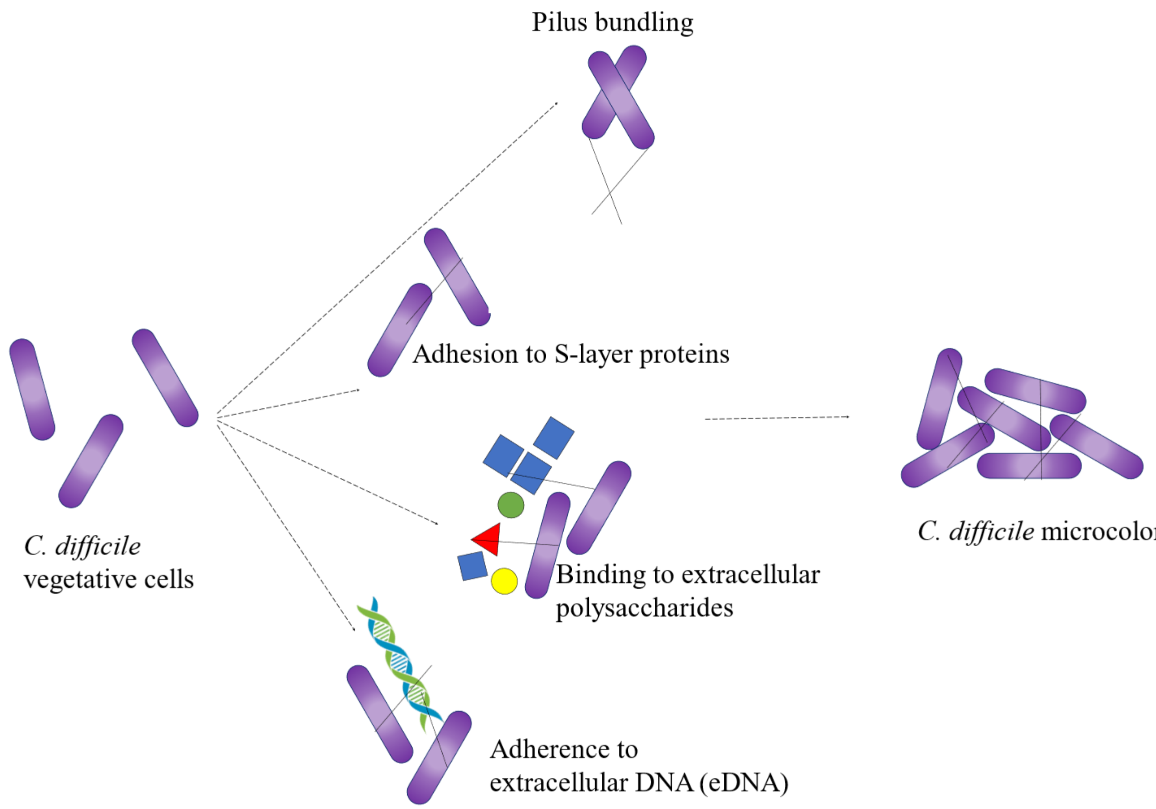

Supporting Figure 8: Schematic of potential mechanisms for T4P-mediated promotion of biofilm formation. Four potential mechanisms of biofilm formation dependent upon type IV pili are depicted; (i) the bundling of type IV pili between adjacent bacterial cells, (ii) the adhesion of T4P to bacterial cell-surfaces, (iii) interactions between T4P and polysaccharides in the extracellular matrix and (iv) recognition of extracellular DNA by T4P.
